# Construction of tumor organoids and their application to cancer research and therapy

**DOI:** 10.7150/thno.91362

**Published:** 2024-01-12

**Authors:** Jiajing Lv, Xuan Du, Miaomiao Wang, Jiacan Su, Yan Wei, Can Xu

**Affiliations:** 1 Institute of Translational Medicine, Shanghai University, Shanghai 200444, China.; 2 Institute of Medicine, Shanghai University, Shanghai 200444, China.; 3 Organoid Research Center, Shanghai University, Shanghai 200444, China.; 4 Biopharma Industry Promotion Center Shanghai, Shanghai 201203, China.; 5 Department of Rehabilitation Medicine, Shanghai Zhongye Hospital, Shanghai, 200941, China.; 6 Department of Orthopedics, Xinhua Hospital, Shanghai Jiao Tong University School of Medicine, Shanghai 200092, China.; 7 Department of Gastroenterology, Changhai Hospital, Naval Medical University, Shanghai 200433, China.

**Keywords:** tumor organoids, cellular components, culture methods, basic cancer research, antitumor therapy

## Abstract

Cancer remains a severe public health burden worldwide. One of the challenges hampering effective cancer therapy is that the existing cancer models hardly recapitulate the tumor microenvironment of human patients. Over the past decade, tumor organoids have emerged as an *in vitro* 3D tumor model to mimic the pathophysiological characteristics of parental tumors. Various techniques have been developed to construct tumor organoids, such as matrix-based methods, hanging drop, spinner or rotating flask, nonadhesive surface, organ-on-a-chip, 3D bioprinting, and genetic engineering. This review elaborated on cell components and fabrication methods for establishing tumor organoid models. Furthermore, we discussed the application of tumor organoids to cancer modeling, basic cancer research, and anticancer therapy. Finally, we discussed current limitations and future directions in employing tumor organoids for more extensive applications.

## Introduction

Cancer has been a significant public health problem worldwide. It is estimated that approximately 1670 people will die of cancer per day in 2023 in the United States 1. Effective treatment remains a significant unmet need for most cancer patients. One of the crucial factors is that extensive inter- and intratumoral heterogeneity makes it extremely difficult to predict successful anticancer therapy 2. Therefore, there is an urgent demand for reliable models that can effectively recapitulate cancer patients' complicated tumor microenvironment (TME) 3.

Unlike conventional long-term used cancer cell line models, patient-derived cancer cells (PDCs) reproduce the molecular properties (e.g., RNA expression and mutations) of the initial primary tumor more precisely 2. Jin-Ku Lee *et al.* have reported a high clinical concordance between PDC-based sensitivities of targeted therapeutics and their clinical response in retrospective studies 2. However, PDC models are oversimplified and fail to recapitulate the original tumors' structural, physiological, and transcriptional characteristics 4. For instance, the 2D cultured hepatocytes quickly lose polarity because of structural distortion within the cell monolayers, leading to their dedifferentiation and death 5. Alternatively, another typical tumor model of patient-derived xenografts (PDXs) can fill the gap established by directly transplanting tumor fragments surgically dissected from cancer patients into immunodeficient mice 6. PDXs preserve the original structure and the genomic and gene expression profiles of primary tumors. However, the mouse-derived matrix almost entirely replaced the primitive human-associated matrix after 3-5 passages, when PDXs can be used for drug screening 7,8. This phenomenon leads to genetic drift and makes the model less accurate 9. Furthermore, low engraftment success rate, too long of a course (generally 4-8 months), and failure to replicate the immune system also restrict its application 6. Accordingly, alternative tumor models are urgently needed to make up for the defects of these traditional models.

Organoids are *in vitro* 3D constructs comprising multiple cell types originating from organ-specific progenitor cells, human stem cells, or disassociated tumor tissues 10. In 2009, Sato and his colleagues established the first adult stem cells (ASCs)-derived organoid, opening a new chapter on organoid development 11. Likewise, Sato *et al.* were the earliest research teams to establish tumor organoids. They successfully cultured adenoma and adenocarcinoma organoids in 2011 by optimizing the previous colon culture systems 12. The *in vitro* 3D culture can partly simulate the TME due to cell-extracellular matrix (ECM) interplay 13. Moreover, it hardly needs to adapt to a new host, avoiding the occurrence of genetic drift. Meanwhile, unlike PDX models, organoids tend to be produced on a large scale for high-throughput drug screening. By modeling cancer more accurately, organoids represent advanced *in vitro* tumor models. As a more cost-effective and faster alternative to PDXs, organoids fill the gap between PDCs and PDXs.

**Figure 1:**
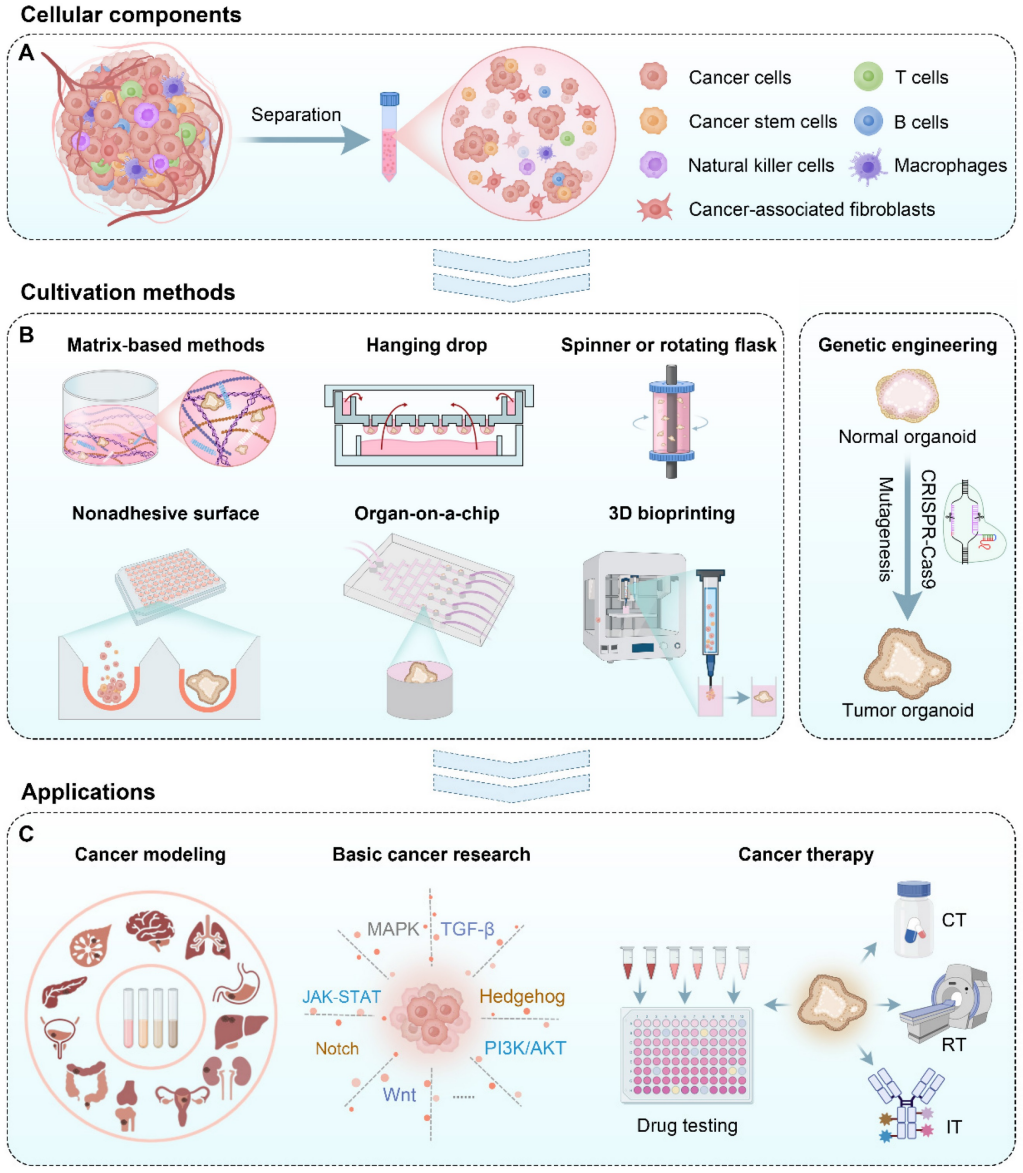
Schematic illustration of cellular materials, various tumor organoid construction methods, and their applications to cancer research and therapy. CT: Chemotherapy; RT: radiotherapy; IT: immunotherapy.

Tumor organoids are commonly generated from two primary sources: induced pluripotent stem cells (iPSCs) and freshly resected tumor tissue 14. Currently, most cell materials are obtained by separating the original tumor tissues into a cell cluster mixture containing tumor stem cells (**Figure 1A**) 15. Then, these cell clusters were suspended in the medium supplemented with particular growth factors and grew into tumor organoids by various methods such as a matrix-based method, hanging drop, rotary system, low-adhesive platform, organ-on-a-chip, 3D bioprinting, and genetic engineering (**Figure 1B**). These formed tumor organoids can be used for cancer modeling, basic cancer research, drug testing, and personalized medicine (**Figure 1C**). In this review, we introduced cellular components and various approaches for tumor organoid generation and elaborated on the application of tumor organoids in cancer research and therapy. Finally, we discussed current challenges and future perspectives for the broader application of tumor organoids.

## Cellular materials for tumor organoid construction

### Commonly used cell materials

Depending on differentiation procedures, pluripotent stem cells-derived organoids contain all the cell types derived from three germ layers, including epithelial and nonepithelial cells. They, thus, are more suitable to study organ development 16. In contrast, commonly established from healthy and diseased tissues, ASCs-derived organoids comprise epithelial cells and can amplify patient-derived cells *in vitro*, representing an excellent model to investigate tissue regeneration and homeostasis 17. The first patient-derived organoids (PDOs) were built from adult human intestinal epithelial cells in 2011 11,12. Then, the protocols were employed to generate organoids from other epithelial tissues, including healthy and diseased 18. Nowadays, ASC-stemmed organoid technology is extensively applied to generate collections of cancer patient-derived tumor cultures (i.e., “living biobanks”) that preserve tumor heterogeneity 19.

Tumor organoids are produced from solid surgical resection materials and smaller solid punch or needle biopsies 20. First, nonepithelial tissues, such as fat or muscle, are removed from the tissue samples to the greatest extent 17. Then, a part of the tumor tissues is preserved for subsequent molecular or biochemical analysis 21,22. Typically, the remaining tissue is minced into tiny fragments of 1-3 mm^3^ with a sterile scalpel, followed by enzymatic digestion until 2-10 cell-containing clusters are observed 17,18. Subsequently, the dissociated single cells or cell clusters are filtered through a cell strainer with pore diameters of 100 μm to get rid of the undigested tissue fragments and seeded in a 3D ECM hydrogel, such as Matrigel, Geltrex, or basement membrane extract (BME) 17,21,22.

After seeding, the cells are cultured in an appropriate medium containing a growth factor cocktail that can stimulate a regeneration response in the stem cells of the epithelium. Crucial components of this cocktail commonly include 1) activators of the Wnt signaling pathway, e.g., the LGR5 ligand R-spondin and the Wnt ligand 23-25; 2) ligands of tyrosine kinase receptors, e.g., epidermal growth factor (EGF), functioning to promote epithelial proliferation 26,27; and 3) inhibitors of bone morphogenetic protein/transforming growth factor-β (TGF-β) signaling, e.g., Noggin, aiming to inhibit epithelial differentiation 28. The initial success rate of tumor organoid culture relies on specific types of tumors. After several weeks of cultivation, tumor organoids are generated partially 17,21,29.

Else Driehuisa *et al.* collected the patient's tumor samples in the distal bile duct and pancreas 20. These tumors were cut into small fragments, digested with collagenase, and sheared with 5 mL pipettes. The obtained single-cell suspension was cultured in the medium supplemented with various growth factors, such as Wnt3a-conditioned medium, 50 ng/mL human EGF, and Noggin for organoid cultivation. The organoids were harvested and disrupted by digestion using TrypLE Express or mechanical shearing. These organoids recapitulated histological and genetic characteristics of original pancreatic ductal adenocarcinoma (PDAC). A panel of 76 drugs were tested in the organoids, revealing sensitivities currently not exploited in the clinical 20.

The overgrowth of normal gastric organoids from gastric cancer (GC) organoids frequently occurs, preventing the buildup of slow-growing GC organoids. To construct GC organoids from clinical samples, Kosaku Nanki *et al.* modified the culture condition based on the dysregulated signals in human GCs, such as the TP53, TGF-β, RHO, and RAS-PI3K pathways 18. They first used an MDM2 inhibitor, Nutlin-3, to enrich TP53 mutant GC organoids. Second, as the recovery of individualized organoid cells requires ROCK inhibition, they exploited a ROCK inhibitor (Y-27632)-free medium to collect RHO-dysregulated GCs. Third, they enriched GC organoids insensitive to TGF-β stimulation by incubating them with TGF-β without A83-01. Finally, they collected organoids with ligand-independent receptor tyrosine kinase (RTK) signal activation by removing EGF and FGF10 from the culture medium. Through the modified condition, the success rate of organoid cultivation was increased from 54.7% (23 lines from 42 specimens) to 74.6% (44 lines from 59 specimens). Moreover, all the organoids could maintain their propagation for at least 3 months.

In addition to epithelial tumors, a mesenchymal tumor organoid was successfully constructed 30. Michael T Meister *et al.* generated a library of 19 pediatric rhabdomyosarcomas (RMS) organoids at a success rate of 41% through the overgrowth of patient-derived tumor cells in the medium, which contained all major subtypes. These organoid models closely recapitulated the parental tumors' genetic, molecular, and histological features.

### Supplementary cellular components

TME is a pivotal player in modifying cancer progression and therapeutic response. A primary impediment restricting the development of cancer treatment is the discrepancy of TME between tumor models and patients. It is challenging to characterize TME because maintaining TME viable in tissue culture and manipulating it *ex vivo* are somewhat tricky 31. The TME components vary across tumor types but generally include stromal cells, vasculature, and ECM 32. Despite possessing a 3D structure, tumor organoids often fail to simulate an intact microenvironment. This defect occurs because exogenous growth factors and small molecules in the culture medium of organoids may lead to clonal selection 33. For instance, Luo *et al.* found that organoid culture poorly supports cancer-associated fibroblast (CAF) viability 34. Accordingly, supplementing tumor organoids with patient-specific TME components is emerging as an appealing strategy to optimize and perfect this model.

CAFs are the most common cell types of stromal cells in the TME. Mesenchymal stem cells (MSCs) are postulated to differentiate into CAFs in the TME 35. CAFs play a crucial role in tumor progression and chemoresistance. Luo *et al.* encapsulated colorectal cancer (CRC) organoids within a well-defined hyaluronan-gelatin hydrogel and co-cultured them with patient-derived CAFs 34. The results showed that the CAFs could maintain the proliferation of CRC organoids even without growth factors. Moreover, the CAF-co-cultured CRC organoids restored various biological pathways of the parental tumors, making them suitable for testing standard-of-care drugs.

**Figure 2:**
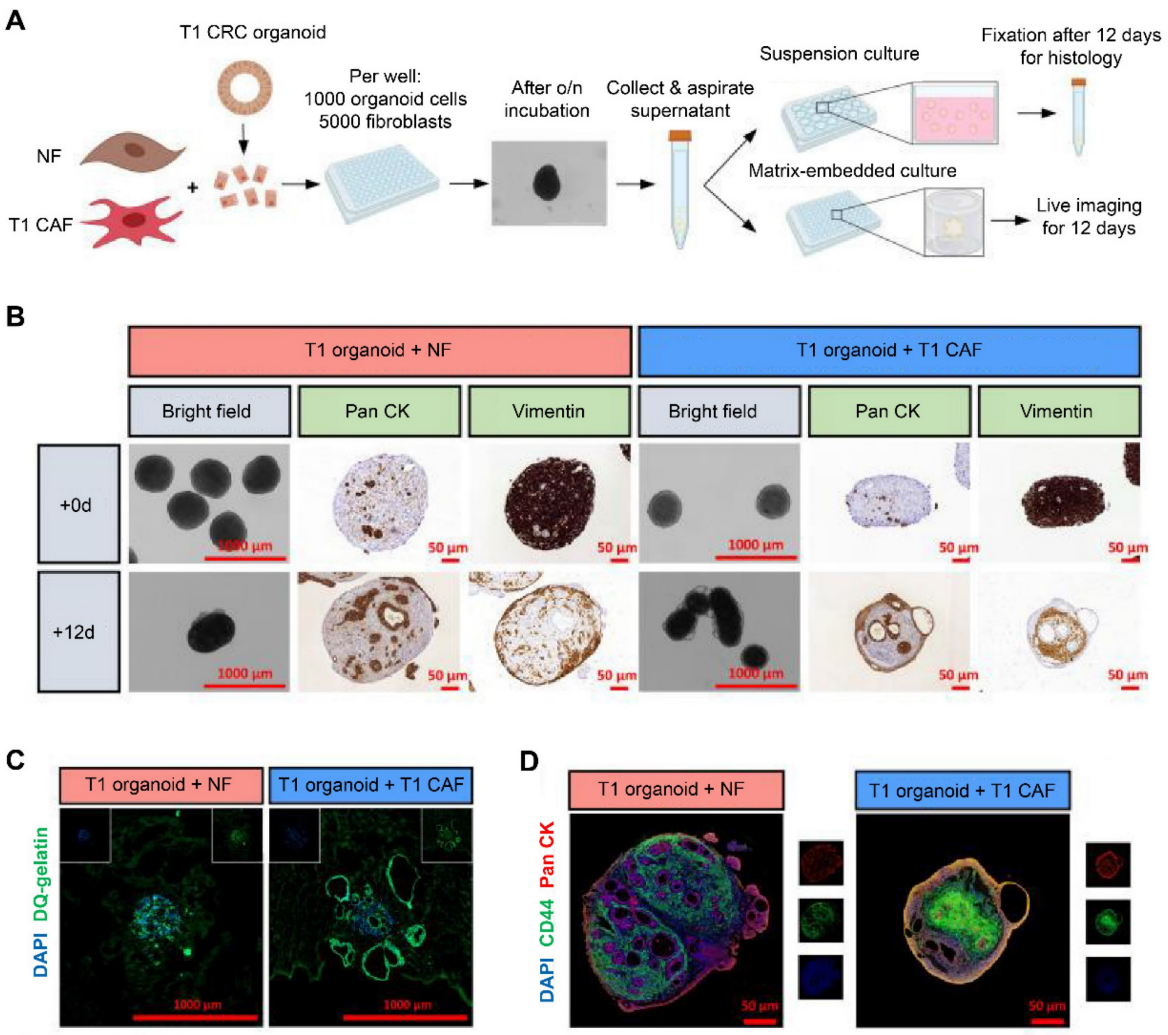
Co-cultures of normal fibroblast (NF)-early-stage cancer-associated fibroblast (T1CAF) pairs and early-stage colorectal cancer (T1CRCs) organoids. (A) Procedures for the co-culture of organoids and fibroblasts. (B) Representative images of suspension co-cultures of T1CRC organoids with unmatched NF-T1CAFs. Epithelial cells and fibroblasts were stained with pan cytokeratin (Pan CK) and vimentin, respectively. (C) *In situ* zymography showing proteolytic activity with dye-quenched gelatin as a substrate on cryosections of Matrigel-embedded co-cultures of T1 CRC organoids with unmatched NF-T1CAFs after 12 days of culture. (D) Immunofluorescence staining of suspension co-cultures for CD44 (green), Pan CK (red), and DAPI (blue). Adapted with permission from 36, copyright 2023, Elsevier Ltd.

Similarly, Dang *et al.* integrated unmatched early-stage CAFs (T1CAFs) and normal fibroblasts into CRC organoids at a 5:1 ratio (**Figure 2A**) 36. After 12 days of suspension culture, normal fibroblasts (vimentin-marked) were still localized on the periphery of the structure (**Figure 2B**). In contrast, T1CAFs had migrated into the core of the co-culture, while cancer cells (pan cytokeratin-labeled) localized on the edge. Furthermore, relative to a normal fibroblast-CRC system, T1CAF-CRC co-culture showed higher proteolytic activity at the outer rim and increased epithelial expression levels of CD44, indicating T1CAF-induced tumor invasion and progression (**Figure 2C-D**). In another study, Schuth *et al.* established 3D co-cultures of primary PDAC organoids and patient-matched CAFs 37. The co-culture system showed upregulation of genes relevant to epithelial-to-mesenchymal transition (EMT), and several potential receptor-ligand interactions associated with EMT were identified, revealing a crucial role of CAF-driven EMT induction in PDAC chemoresistance.

CAFs can also be derived from human iPSCs. Kenta Takeuchi *et al.* co-cultured patient-derived PDAC cells with human iPSC-derived mesenchymal cells and vascular endothelial cells in an air-liquid interface (ALI), creating a fused PDAC organoid 38. The organoids were further induced to recapitulate two statuses of PDAC. The quiescent organoids were drug-resistant due to the desmoplastic stroma secreted by the multiple types of CAFs derived from human iPSCs. The proliferative organoids re-proliferated after chemotherapy and could be used to study tumor recurrence.

The quality and magnitude of natural killer (NK) cells, T cells, macrophages, and, more recently, B cells within the TME have decisive effects on the outcome of immune therapy 39. Neal *et al.* developed an ALI method to successfully expand PDOs from mouse tumors in syngeneic immunocompetent hosts or > 1000 biopsies as native immune cells-embedded tumor epithelia (T, B, NK, macrophages) 40. Tumor-infiltrating lymphocytes in the PDOs accurately retained the original tumor T cell receptor spectrum. Furthermore, human and murine PDOs successfully recapitulated immune checkpoint blockade. The organoid-based expansion of primary tumor epithelium en bloc containing endogenous immune stroma contributes to immuno-oncology investigation within the TME and personalized immunotherapy testing.

In addition, it is a more straightforward method to co-culture organoids with immune cells directly. Cattaneo *et al.* generated and assessed tumor-reactive T cells by coculturing tumor organoids and autologous peripheral blood lymphocytes 41. After 2 weeks of culture, CD8^+^ T cell populations were obtained from ~33-50% of samples from patients with CRC and non-small-cell lung cancer. This co-culture system enables the test of T-cell-based immunotherapy *ex vivo* at the individual patient level. Similarly, Zhuolong Zhou *et al.* constructed a T cell-engaging PDAC organoid platform by two-step cell packaging 42. They first generated PDAC organoids from KPC (*LSL-Kras^+/G12D^; LSL-Trp^53+/R172H^; PDX1^-Cre^*) tumor-bearing mice, which included epithelial tumor cells, CAFs, vascular endothelial cells, and macrophages. Then, they packaged the outside Matrigel layer of the organoids with T cells derived from OT-I transgenic mice that recognized antigens presented by KPC tumor cells. The tumor organoids recapitulate the cell composition and histological structure of primary PDAC tumors, enabling the investigation of T cell infiltration and cytotoxicity within the desmoplastic and immunosuppressive tumor microenvironment. Through the PDAC organoid platform, epigenetic inhibitors I-BET151 and ITF2357 were screened out, which exhibited impressive antitumor efficacy in combination with anti-PD-1 therapy.

Macrophage infiltration plays a crucial role in PDAC progression 43. Shengwei Jiang *et al.* constructed a macrophage-organoid co-culture model at a cell density ratio of 1:3 of macrophages to PDAC cells 44. They found that macrophage-secreted CCL5 could activate the CCR5/AKT/Sp1/CD44 axis, endowing PDAC cells with stemness and chemoresistance; PDAC cell-derived AREG promoted macrophage cells to secret CCL5 *via* the Hippo-YAP pathway. Mithramycin could magnify the antitumor efficacy of gemcitabine by targeting the feedback loop. Notably, the data from the PDOs were corroborated with the clinical data 44.

Joanne Tze Chin Lim *et al.* established endothelial cell-co-cultured hepatocellular carcinoma (HCC) PDOs by coculturing PDX-derived organoids with human umbilical vein endothelial cells (HUVECs) 45. The PDOs reproduced known angiocrine signaling, and the endothelial cells led HCC cells to create an inflammatory microenvironment by recruiting immune cells. The macrophages were polarized toward a pro-angiogenic and pro-inflammatory subset, resembling a tumor-associated macrophage phenotype as previously described in HCC. These characteristics made the co-culture models suitable for understanding and targeting the interactions between the immune niches and angiogenesis. In addition, some researchers have combined endothelial cells and other types of cells, such as mesenchymal cells and immune cells, for tumor organoid co-culture 38,46,47. Various cell materials additionally supplemented for tumor organoid co-culture are summarized in **Table 1**.

## Fabrication methods for tumor organoids

In contrast to tumor organoids, tumor spheroids are a simpler 3D tumor model generally obtained from single-cell suspensions that self-aggregate or are forced to aggregate 51. Currently, there are many methods for spheroid cultivation, such as hanging drop, rotating flask, and nonadhesive surface. These methods can also be used to culture tumor organoids 52. Subsequently, the methods used for tumor organoid construction are introduced in detail.

### Matrix-based methods

Matrigel is the most extensively used natural ECM. However, its employment is restricted by some deficiencies, such as batch-to-batch variations and potential pathogen contamination. Accordingly, decellularized ECMs and natural polymer-based matrices have been developed as an alternative to Matrigel. In addition to these natural ECMs, synthetic ECMs with defined chemical components and physical properties have attracted extensive attention.

#### Natural matrices

Natural matrices include Matrigel, decellularized ECM, and natural polymer-based matrices 53. For example, hyaluronic acid (HA), gelatin, collagen, fibronectin, chitosan, alginate, cellulose, and glycidyl methacrylate-HA have been extensively used as the main constituents of current natural matrices for organoid culture 54.

Currently, the cultivation of most organoids depends on Matrigel, a commercialized matrix comprising collagen type Ⅳ, laminin, and growth factors 55. Many tumor organoids of colon cancer 56, rectal cancer 57, and pancreatic cancer 58 have been successfully cultured with Matrigel. However, Matrigel has some undeniable defects. First, since Matrigel extracted from the Engelbreth-Holm-Swarm mouse sarcoma is a raw material, it exhibits sizeable batch-to-batch variability. For example, on average, Bi *et al.* identified 956 proteins from Matrigel samples, and as many as 1637 proteins were detected from three Matrigel samples 59. In addition, given its mouse origin, Matrigel can potentially carry pathogens to infect macrophages and affect the immune systems 55.

**Figure 3:**
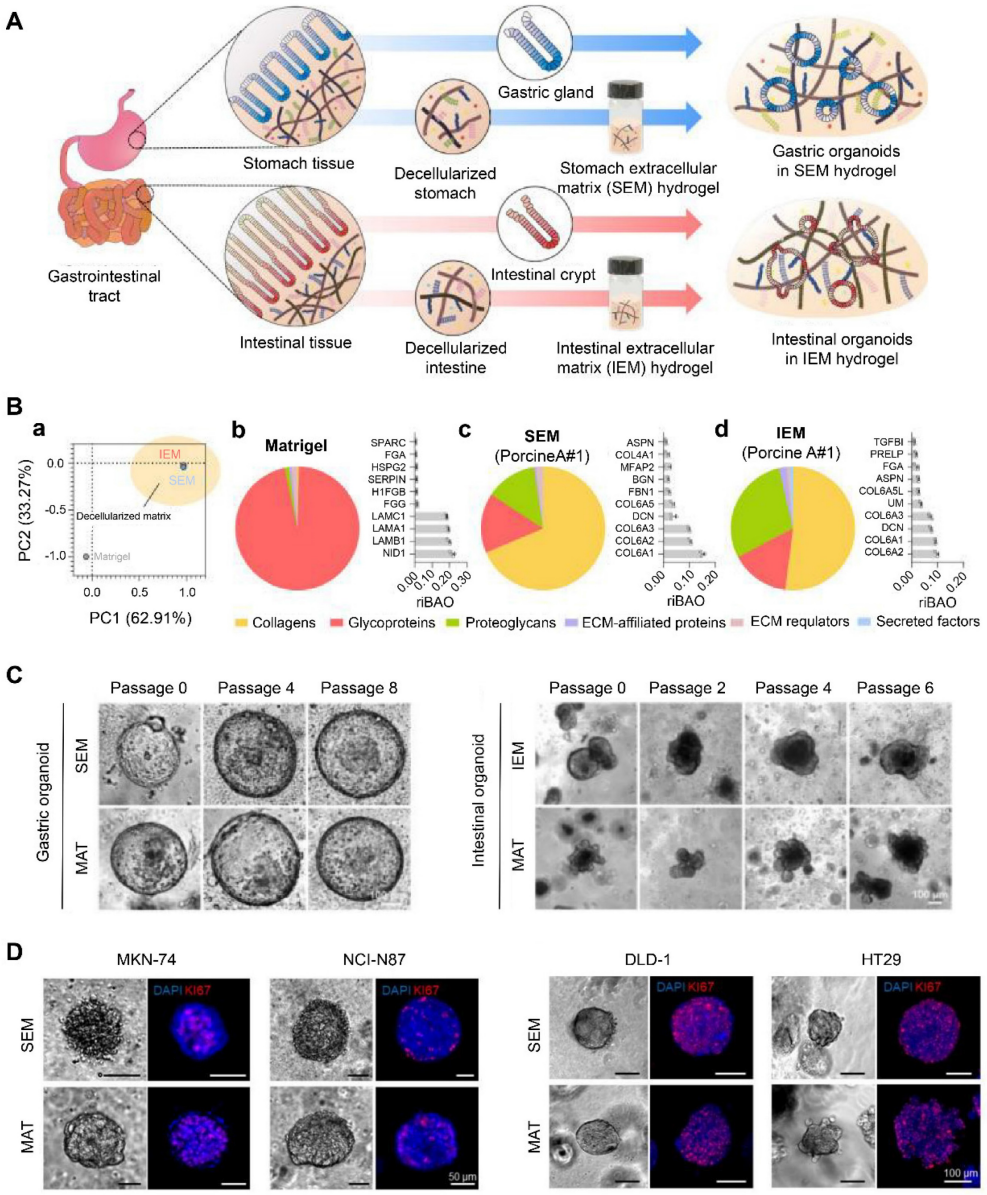
Gastrointestinal (GI) tissue-derived extracellular matrix (ECM) hydrogels *via* decellularization for organoid culture. (A) Schematic illustration of the preparation of GI organoids using ECM hydrogels (stomach extracellular matrix (SEM) and intestinal extracellular matrix (IEM)) derived from the decellularized GI tract. (B) (a) A principal component analysis (PCA) of matrisome proteins existing in Matrigel, SEM, and IEM. All the protein composition and the most abundant top 10 matrisome proteins in (b) Matrigel, (c) SEM, and (d) IEM. (C) Representative brightfield images showing (left) gastric and (right) intestinal organoids cultured in SEM/IEM hydrogel and Matrigel (MAT) at various passages. Scale bar: 100 μm. (D) Representative immunofluorescence images of GI tumor organoids derived from GI cancer cell lines. Adapted with permission from 55, copyright 2022, Nature Publishing Group.

Decellularization is a process that removes cells while preserving ECM composition and structure. Therefore, it can retain crucial features of the original tissue, such as desmoplasia and stiffness 60. Kim *et al.* developed ECM hydrogels derived from decellularized gastrointestinal tissues (**Figure 3A**) 55. The tissue-specific proteome components were preserved in these hydrogels, distinct from Matrigel (**Figure 3B**). These decellularized hydrogels could maintain the long-term growth of gastrointestinal organoids and facilitate the establishment of gastrointestinal tumor organoids due to their specific gastrointestinal microenvironment, representing an appealing alternative to Matrigel (**Figure 3C-D**) 55. Peritoneal metastases of CRC are associated with poor survival. Varinelli *et al.* found that decellularized ECM of the peritoneal cavity could maintain the growth of organoids derived from peritoneal metastases, and these *in vitro* 3D models preserved the characteristics of *in vivo* peritoneal metastases 61. In addition, Tienderen *et al.* established two types of cholangiocarcinoma organoids using decellularized tumor or liver scaffolds 60. They found that the transcriptome of cholangiocarcinoma organoids in a tumor-derived ECM resembled that of* in vivo* parental tumor tissues more than the organoids maintained in liver-derived ECM.

Natural polymers are compatible and exhibit a structure resembling natural ECM. Lingling Ou *et al.* constructed melanoma PDOs by embedding them into matrices: Matrigel or collagen gel 62. They found that melanoma PDOs cultured in collagen gel had immune cell components and morphology similar to the parental tumors, as Matrigel did. These results supported the applicability of collagen matrix for organoid culture. Specific matrix characteristics, such as adhesion specificity and stiffness, affect tumor organoid generation 63. For example, Bordeleau *et al.* reported that increasing stiffness of collagen-based matrix, irrespective of matrix density, promoted tumor vasculature formation 64.

Another advantage of natural polymers is that their biochemical and biophysical properties are adjustable *via* chemical modification, such as conjugating cell adhesive peptides and cross-linkable groups. Shengyong Ng *et al.* synthesized HA-phenol (HA-Ph) and gelatin-phenol (gelatin-Ph) conjugates and covalently crosslinked these conjugates through hydrogen peroxide and horseradish peroxidase catalysis 65. They found that gelatin-Ph hydrogels supported CRC organoid growth better than gelatin-Ph/HA-Ph or HA-Ph hydrogels. Moreover, high matrix stiffness combined with hypoxia promoted the growth and metabolism of the CRC organoids. These biochemically and mechanically defined matrices synthesized *via* enzyme crosslinking showed desirable properties for tumor organoid culture. In addition, considering that the CRC ECM is enriched in collagen I and HA, Xiaobei Luo *et al.* prepared HA-gelatin hydrogels to replace traditional basement membrane extracts by conjugating thiol-modified HA to thiol-modified gelatin *via* a polyethylene glycol (PEG)-diacrylate linker 34. They constructed CAF-co-cultured CRC PDOs with the hydrogel and found that even without growth factors, CAFs could support the growth of the CRC PDOs in the hydrogels. Moreover, the co-cultured PDOs recapitulated various biological pathways of parental tumors, making them suitable for drug testing.

#### Synthetic matrices

Besides natural matrices, synthetic polymers are also extensively investigated for tumor organoid culture because they can be chemically defined and have low batch-to-batch variability 47. PEG is one of the most extensively used synthetic polymers for organoid culture because its unique chemical structure allows versatile and controllable modifications. Tian *et al.* engineered 3D tumor organoids by modifying the PEG-MAL hydrogels with adhesive ligands with different integrin specificities 66. The hydrogel presented defined densities of ligands to integrins on follicular dendritic cells and lymphoma cells, enabling the reproduction of the lymphoma-associated TME and exploring associated chemotherapeutic resistance. Similarly, Mosquera *et al.* constructed a synthetic four-arm PEG-4MAL hydrogel to propagate patient-derived prostate tumor organoids. The hydrogel was tunable by conjugating different ECM peptide mimics, such as collagen-mimicking GFOGER peptide and fibronectin-mimicking REDV peptide 25. The ECM types considerably affected the response of the PDOs to small-molecule inhibitors of dopamine receptor D2 (DRD2) and epigenetic targets. Finally, they found that the therapeutic response in prostate cancer with drug-resistant ECM was improved by first cellular modeling with epigenetic inhibitors, followed by DRD2 treatment. These findings in the PDOs of prostate cancer facilitated the development of new therapies to address their drug resistance.

Thermosensitive hydrogels and hybrids of synthetic and natural polymers are also commonly used matrices. For example, Poly (N-isopropylacrylamide-coacrylic acid) (PNIPAM-AA) is a thermosensitive hydrogel that can undergo sol-gel transformation at specific temperatures for cell loading 67. Ehsan Atefi *et al.* prepared hybrid materials comprising an aqueous two-phase dextran and PEG 68. When a submicroliter drop of the dextran phase containing cells was submerged into a PEG bath, a round drop formed that confined cells to generate a spheroid spontaneously.

### Hanging drop

Hanging drop is a culture method whereby cells are attached to a coverslip and bathed in a drop of a specific culture medium 23,69; then, the coverslip is inverted and sealed to a microscope slide to hang the drop. Cells will aggregate slowly in the bottom center of the droplet and eventually form organoids (**Figure 1B**). This culture method has many advantages, such as maintaining 3D tissue architecture, requiring only a tiny amount of medium, and facilitating efficient gas exchange 70. Moreover, these cultures notably facilitated preserving *ex vivo* signaling activity and functional integrity 70. The dimension of organoids can be adjusted by controlling the droplet volume or cell suspension density.

By this technique, Djomehri *et al.* realized the high-yield generation of highly spherical large breast organoids (~1 mm diameter) in a one-drop-one-organoid format 27. The scaffold-free organoid model with high reproducibility and consistency can detect cellular collagen Ⅰ generation without noise from exogenous collagen and can receive various stimuli from the exogenous treatments or microenvironment while avoiding matrix binding. This method also generated organoids from primary metaplastic mammary carcinomas, preserving the primary tumors' high-grade spindle cell morphology 27. In addition, Eder *et al.* employed the 3D hanging drop technique to successfully establish prostate cancer organoids as tumor epithelial monocultures and epithelial-stromal co-cultures on 96-well plates 26.

### Spinner or rotating flask

**Figure 4:**
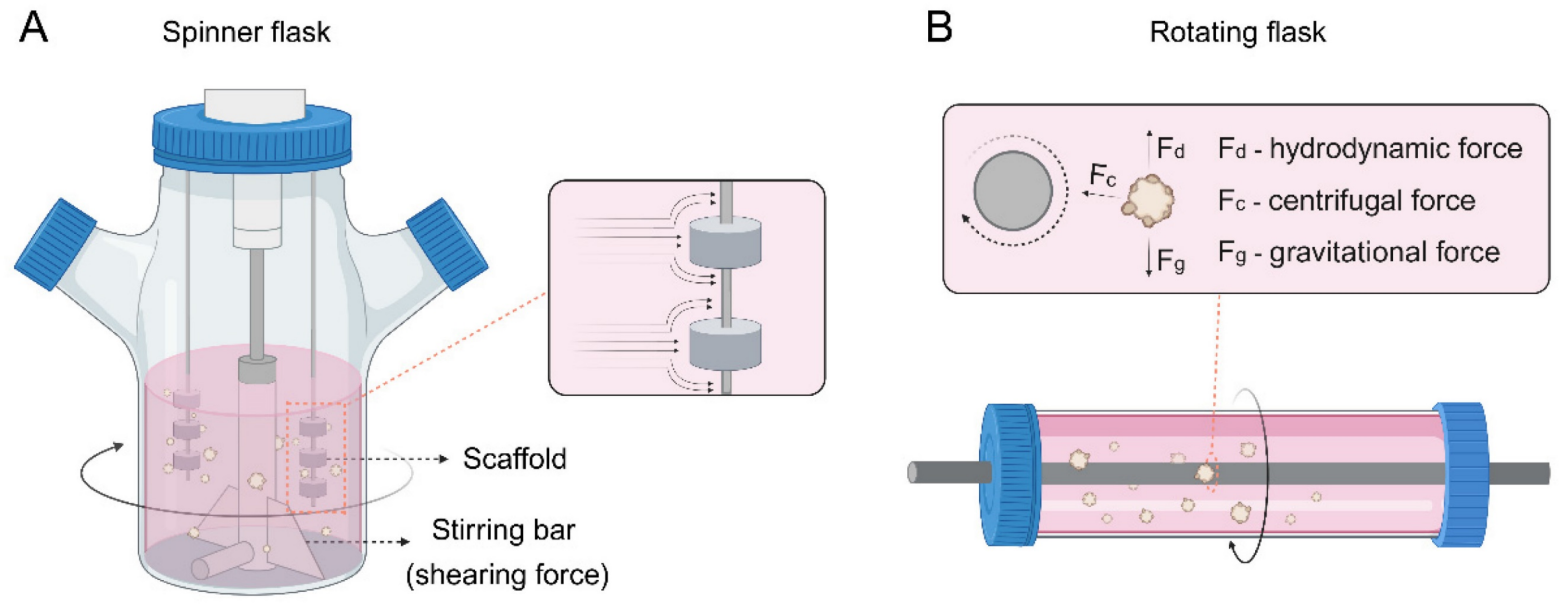
(A) Spinner or (B) rotating flasks for organoid construction.

Spinner or rotating flasks are generally used for large-scale generation of tumor spheroids or organoids, where cells are cultured as multicellular aggregates in stirred suspension culture (**Figure 1B**) 71. The spinner flasks contain a magnetic stirrer at the center of the flask that continuously distributes nutrients and O_2_ throughout the medium (**Figure 4A**). Stationary scaffolds are fixed and suspended through a rod inside the flask, and the cells can flow across the surface of the scaffolds. However, cells undergo shearing force caused by the continuous stirring, which is adverse to cell physiology 72. In contrast, rotating flasks exert upward hydrodynamic force by rotating the flask itself, which counterbalances downward gravitational force and thus leads the cells to suspend (**Figure 4B**) 73. This approach subjects cells to less shearing force than spinner flasks, providing a milder environment for cell growth. In addition, the volume of tumor spheroids or organoids can be adjusted by changing the rotational speed of the flasks.

Schneeberger *et al.* exploited spinner flasks to expand many human liver organoids 74. Since oxygenation was improved in the spinner flasks, organoids rapidly grew and achieved a 40-fold cell propagation on average after 2 weeks, compared to a 6-fold propagation in static cultures. Furthermore, differentiation in the spinner flasks led to highly upregulated mature hepatocyte markers relative to static organoid cultivation, which lays the foundation for organoid application for tissue engineering and liver transplantation.

Yang *et al.* produced testicular organoids with testicular cells by combining a hanging drop and a rotation system 75. They reported that testicular cells could spontaneously form organoids with tubule-like structures *via* hanging drop. These established organoids showed similar gene expression to adult testis tissue, exerted testosterone with preserved gonadotropin responsiveness, and were sensitive to reproductive toxicants. This organoid helps explore the self-organization process of testicular cells and serves as an experimental model for pharmacotoxicology testing, reproductive biology research, and regenerative medicine research.

### Nonadhesive surface

A nonadhesive surface is a traditional and the most straightforward method for cultivating tumor spheroids or organoids 76. Cell-matrix interaction is interrupted when growing on a nonadhesive or poorly adhesive surface, and cell-cell interplay is privileged. Therefore, cells tend to aggregate into spheroids under such conditions (**Figure 1B**) 76. The commonly used nonadhesive surfaces include HA-based surfaces, bovine serum albumin-modified surfaces, and poly(N-isopropyl acrylamide) (PNIPAAm)-based porous hydrogel 77-79. Simple as the method is, there are still some drawbacks. Controlling the size and tracking each spheroid's growth is hard during culturing.

Concave microwells with nonadhesive surfaces were utilized for spheroid or organoid culture 80. When tumor cells are planted into concave microwells, of which the surfaces resist cell adherence, tumor cells will attach and form a multicellular aggregate, i.e., spheroids or organoids. The volume of the spheroid can be adjusted by changing the seeding amount of cells and the dimension of concave microwells 81. The status of spheroids can be monitored by traditional tools such as a bright field and fluorescent microscope, and the spheroids can be labeled with fluorophores to visualize the cell cytoskeleton 82.

**Figure 5:**
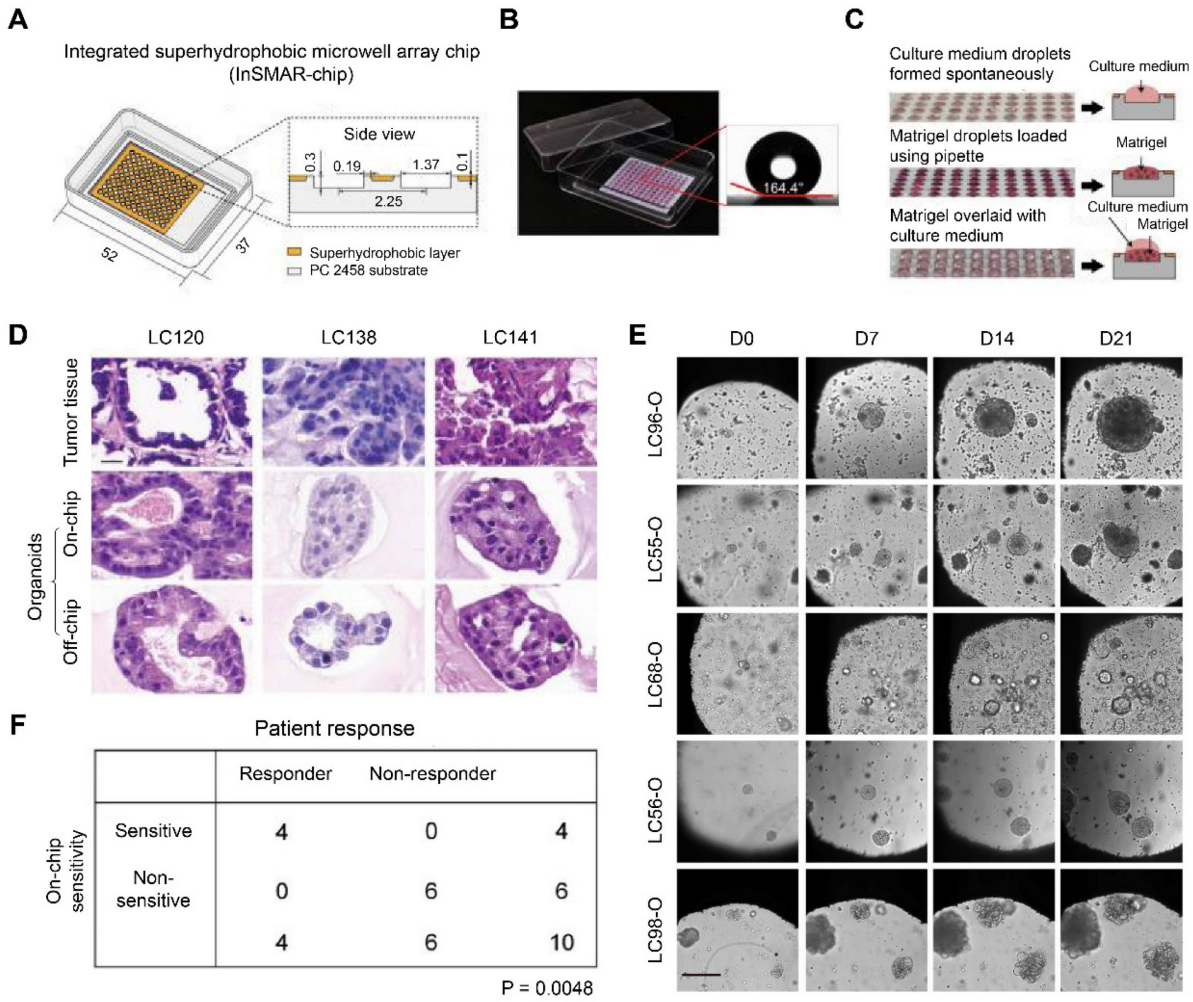
Construction and characterization of the integrated superhydrophobic microwell array chip (InSMAR-chip). (A) Schematics (left) and cross-section view (right) of the InSMAR-chip. (B) Photograph of an InSMAR-chip with a droplet array in the microwells. The contact angle of the superhydrophobic surface is > 160°. (C) Photographs of the droplet array in the microwells. (Top) When the excess medium was removed from the chip, the droplet array of culture medium formed spontaneously. (Middle) The droplet assay of the Matrigel loaded in the microwells. (Bottom) The Matrigel droplets are overlaid on the culture medium via the spot-cover method. (D) Representative H&E staining images of the parental tumor tissue and the corresponding LCOs cultured on the InSMAR-chip (on-chip) and the traditional multiwell plate (off-chip). Scale bar: 20 µm. (E) LCOs maintain continuous growth on the InSMAR chip for 21 days. Scale bar: 200 µm. (F) Correlation between patient response and on-chip drug sensitivity. Adapted with permission from 83, copyright 2021, Nature Publishing Group.

Hu *et al.* designed an integrated superhydrophobic microwell array chip (InSMAR chip) to replace the traditional 96-well microplates for culturing lung cancer organoids (LCOs) to speed up the process of drug sensitivity tests. The 100-µm recessed top surface of the microwell array was covered with a layer of a home-made superhydrophobic paint bearing titanium dioxide (TiO_2_) nanoparticles in ethanol-based perfluorooctyltriethoxysilane suspension (**Figure 5A**). Since the superhydrophobic surface caused a robust repelling force with a contact angle > 160°, the culture medium can spontaneously form a uniform droplet array in the microwells after removing the excessive medium (**Figure 5B**). In addition, the Matrigel solution bearing a small number of organoids can be quickly loaded into each microwell with an electronic pipette operated in the multi-disperse mode, forming a uniform droplet array (**Figure 5C**). After the Matrigel solution was gelled, 2.4 µL of culture medium was covered onto each droplet to promote organoid growth. LCOs cultured on the InSMAR chip maintained the 3D structures of the original tumor tissue and continuously grew for over 3 weeks (**Figure 5D-E**). Finally, the 1-week drug sensitivity results in the LCOs were highly consistent with the clinical data with 100% specificity and accuracy (**Figure 5F**) 83. Similarly, Jung *et al.* described a scalable organoid production platform containing 8-well strips and 8 × 9 microwells (500 µm) per strip to cultivate organoids from CRC tissues 84. The platforms were precoated with 10% bovine serum albumin in PBS to endow the bottom of microwells with low attachment. Then, single cells separated from patient CRC tissues were seeded at a density of 100 cells/microwell and enabled suspension cultivation in a complete culture medium containing 2% Matrigel. The platform can simultaneously generate 864 organoids, indicating a vast potential in high-throughput assays.

### Organ‐on‐a‐chip

The current versions of tumor organoids have apparent limitations and thus only partially recapitulate disease processes 85. First, tumor organoids usually consist of only epithelial cells and progenitor cells without blood vessels, innervation, immune cells, and other nonparenchymal cells, such as endothelial cells and fibroblasts 86. Second, tumor organoids usually recapitulate tumors in a single organ but cannot mimic cancer metastases in the multiorgan. Organ-on-a-chip can be defined as microfabricated cell culture devices containing microstructures, ECM, and cells, which are developed to recapitulate the functional units of human organs *in vitro* (**Figure 1B**) 87. The recent tendency in the synergistic employment of organoids with organ-on-a-chip allows the establishment of more sophisticated tumor models to study tumor multiorgan metastasis and tumor-stroma interactions 86.

Organ-on-a-chip has shown significant advancement in engineering perfused vascular networks into 3D tumor organoids, creating a more physiology-associated environment *in vitro*. Shirure *et al.* created a quiescent perfused 3D microvascular network with optically clear polydimethylsiloxane (PDMS) before loading patient-derived tumor organoids or cells in a neighboring compartment 88. Nutrients and drugs can be delivered to tumors through the vascular network. As such, primary breast tumor organoids continuously grew for several weeks, leading to robust sprouting angiogenesis. The platform enables the dynamic and synchronous visualization of tumor progression's hallmark characteristics, including cell proliferation and migration, tumor cell intravasation, and angiogenesis. In another study, by integrating 3D tissue with a vascular network, Nashimoto *et al.* established an open-top microfluidic device with an electrochemical sensor to analyze oxygen metabolism 89. They found that the sensor could monitor the change in oxygen metabolism within a patient-derived tumor organoid in a noninvasive, quantitative, and real-time manner.

In addition, multiple organ-on-a-chip systems are being developed to study tumor metastasis. Xu *et al.* constructed a four-organ microfluidic chip that closely recapitulated the *in vivo* microenvironment of lung cancer metastasis 90. This microdevice comprised an upstream “lung” and three downstream “distant organs,” including bone, brain, and liver. The biomembrane separated bronchial epithelial, mononuclear, microvascular endothelial, lung cancer, and fibroblast cells when they grew in the upstream “lung.” Meanwhile, osteocytes, astrocytes, and hepatocytes grew in distant chambers to replicate lung cancer cell metastasis to the bone, brain, and liver, respectively. After culture in this system, lung cancer cells grew into a “tumor mass,” generating metastases to the brain, bone, and liver and damaging astrocytes, osteocytes, and hepatocytes. Furthermore, the metastatic profile in the organ-on-chip system was validated in a nude mouse model, indicating successful replication of *in vivo* microenvironment of tumor metastasis.

Apart from the abovementioned superiorities, organ-on-a-chip can reproduce biophysical factors in the TME. Fang *et al.* designed a microdevice on a microfluidic chip to model the peristalsis in human intestines 91. The chip comprised hundreds of lateral microwells surrounded by a surrounding pressure channel. Human CRC organoids localizing in the microwell were cyclically contracted by pressure channel, recapitulating the mechano-stimulus caused by intestinal muscles. Interestingly, ellipticine-loaded micelles showed decreased internalization in the organoids under peristalsis, leading to suboptimal antitumor efficacy. The results underline that mimicking mechanical stimuli in the physiological environment is crucial when establishing *in vitro* CRC organoids to assess nanomedicine.

### 3D bioprinting

3D bioprinting represents a scaffold-based technique by printing cells embedded into designed bioinks into the desired shape (**Figure 1B**) 92. Bioinks mainly comprise aqueous hydrogel precursor formulations 93. The printability and the capability of maintaining the desired shape postprinting are vital considerations when designing a bioink 94. However, these properties often conflict with the need to maintain the survival and functionality of embedded cells. As a suitable chamber for cells to survive, hydrogels are typically characterized by low elastic modulus and high compatibility with cell-driven remodeling 93.

For high-throughput bioprinting of tumor organoids, one hurdle is that printed volumes take the risk of contacting the sides of wells. As such, surface tension leads bioinks to fall flat, eventually forming 2D structures. To address the problem, Clark *et al.* developed an organoid immersion bioprinting technique by printing brain tumor organoids into support baths (i.e., HA solution) in plates. The baths prevent organoids from reaching the well walls 95. The bioprinting methodology shows excellent potential to produce tumor organoids automatically and high-throughput.

Collagen is the main ECM component of solid tumors, but low viscosity restricts its use in 3D bioprinting 96. Wen Shi *et al.* prepared low-concentration collagen I-based bioinks and used physically crosslinked silk fibroin hydrogel as the support bath 97. After optimized with a thermosensitive HA-based polymer (i.e., HA-poly(N-isopropylacrylamide)), the bioinks could maintain the phenotypes of CAFs and breast cancer of different subtypes. The mouse breast tumor organoids printed with this bioink could recapitulate *in vivo* tumor morphology and cell phenotypes.

3D bioprinting can also be employed to establish 3D biomimetic microvascular constructs. In the abovementioned study, Wen Shi *et al.* bioprinted normal fibroblast cells and HUVECs in the outside region surrounding the inner core region comprising the bioprinted tumor cells 97. The fibroblasts and 3 mg/mL fibrin were supplemented in the bioink for the stromal cells to promote vessel formation. After 7 days of culture, HUVECs formed more capillary-like structures under hypoxia than normoxia.

Alessandro Enrico *et al.* developed a conceptually new method, i.e., cavitation molding. The method exploited cavitation caused by femtosecond infrared laser pulses to restructure collagen hydrogels non-ablatively, creating stable microchannels with diameters ranging from 20 to 60 µm 98. This approach minimized the heat-radiated scope and the mechanical stress on the cells, thus hardly affecting the viability beyond the lumen. Finally, cultivating endothelial cells within these microchannels formed artificial microvasculature.

Willie Wu *et al.* developed a fugitive ink comprising an aqueous solution of a diacrylate-functionalized Pluronic F127 and Pluronic F127 at varying concentrations as the physical gel reservoir and fluid filler, respectively 99. Through this system, they established 3D biomimetic microvascular networks in which two large parent channels are branched to many smaller microchannels. Based on this earlier work, David B. Kolesky *et al.* further printed vascularized, heterogeneous cell-laden tissue constructs 100. They first designed a bioprinter with four independently controlled printheads, by which four PDMS-containing inks are co-printed in a predetermined sequence to generate a heterogeneous 3D structure, with each layer presenting high-aspect-ratio borders. They utilized previously developed Pluronic F127 ink to assemble embedded vasculature due to its easy printing and removal under mild conditions. The resulting tissue constructs were replete with perfusable vessels lined with HUVECs, ECM, and multiple types of cells. Similarly, Weitao Jia *et al.* designed a cell-responsive bioink comprising sodium alginate, gelatin methacryloyl, and 4-arm poly(ethylene glycol)-tetra-acrylate (PEGTA) 101. The hybrid bioink was first crosslinked by calcium ions, followed by the covalent photo crosslinking for 4-arm PEGTA and GelMA to form stable constructs. The bioink sustained the spreading and growth of embedded stem and endothelial cells, generating biologically relevant, highly organized, perfusable vasculature.

Mollica *et al.* prepared decellularized mammary ECM extracts that spontaneously form hydrogels 102. The ECM hydrogels preserved unique structural and signaling profiles and thus led to distinct responses when breast cancer cells and normal mammary cells were cultivated within them. Moreover, they combined the mammary-derived hydrogel with 3D bioprinting to successfully generate large organoids/tumoroids. These findings verified that a tissue-specific ECM with particular properties can be used as a bioink.

Many researchers also culture primary tumor cells through 3D bioprinting 103,104. Tumor organoids and primary tumor cells are derived from patients' tumor tissues and can form 3D structures through 3D printing. However, the most significant difference is their cultural methods. Unlike primary tumor cell culture, tumor organoid culture requires a cocktail of growth factors that stimulate a regeneration response in the stem cells of the tumor epithelium 105. Accordingly, tumor organoids show self-renewal features through tumor progression and re-proliferation, and their differentiation capability allows for recapitulating the unique features of tumor tissues, such as intratumoral heterogeneity 106. Therefore, tumor organoids can better mimic parental tumor tissues' genetic and histological characteristics.

### Genetic engineering

Tumor organoids can also be constructed by introducing tumorigenic mutations into human organoids *via* a gene-editing technique (**Figure 1B**) 107. In contrast to tumor tissue-derived organoids, this type of tumor organoids mimic *in vivo* structural organization to a certain extent. They comprise both normal and tumor cells, enabling the investigations of interactions between nontransformed and transformed cells 107. In addition, this gene-editing model allows for simultaneous analysis of antitumor activity and accompanied toxicity in the same system for drug screening. However, like most organoids, the gene-edited organoids also lack vasculature.

Over the past decade, the gene-editing toolbox has rapidly expanded due to the discovery of the novel, versatile, and easy-to-use clustered regularly interspaced short palindromic repeats (CRISPR)/CRISPR-associated protein (CRISPR-Cas) system 108. Considering the promises of both organoids and CRISPR-Cas system, it is no wonder that these techniques collide. Although various types of CRISPR-Cas systems have been developed, the most extensively used system in human cells is CRISPR-Cas9 due to its high simplicity and efficiency and multiplexed genome editing 108.

Shan Bian *et al.* constructed a neoplastic cerebral organoid by overexpressing the oncogene *MYC* into cerebral organoids through CRISPR-Cas9- and transposon-mediated mutagenesis 107. The neoplastic cerebral organoids exhibited histopathological features, transcriptome signatures, and cell identities resembling the human central nervous system primitive neuroectodermal tumor (CNS-PNET). In particular, overexpression of *MYC* alone was enough to initiate CNS-PNET-like neoplasm in cerebral organoids quickly. In contrast, animal models generally required additional gene editing, such as the silence of p53 and longer experimental times 109. Most importantly, a unique feature of the model is that tumors are initiated by introducing genetic mutations into a tiny portion of cells in the cerebral organoid 107. These mutations simulated human tumor initiation and led to a mixed structure containing normal and tumor cells. Therefore, these organoid models are suitable for investigating tumor biology and assessing drug efficacy in specific DNA aberrations.

By editing mouse fallopian tube epithelial organoids with lentiviral gene transduction and CRISPR/Cas9 mutagenesis, Zhang *et al.* constructed multiple high-grade serous tubo-ovarian carcinoma (HGSC) models 110. These organoids showed mutational combinations similar to those in HGSC patients. The tumorigenic organoids exerted variable responses to chemotherapeutics and created regulatable immune microenvironments by neutralizing organoid-secreted cytokines/chemokines. These findings allowed the development of a chemotherapy/immunotherapy regimen.

ARID1A mutations were extensively observed in human cancer, whereas oncogenic outcomes of ARID1A in human cells are poorly understood. By knocking out ARID1A in primary TP53-/- human gastric organoids with CRISPR/Cas9, Yuan-Hung Lo *et al.* induced tumorigenicity, dysplasia, and mucinous differentiation 111. They identified different pathways downstream of ARID1A mutation, indicating the usefulness of organoid-based genetic cancer analysis in human cells. Similarly, Thege *et al.* induced Myc activation in primary pancreatic organoids *in vitro* using CRISPR activation (CRISPRa) technology 112. These organoids showed increased tumorigenic potentials when inoculated orthotopically *in vivo*. Moreover, Myc activation resulted in an immune-suppressive “cold” TME. These findings reveal that CRISPRa is valuable for the rapid function identification of putative oncogenes.

Conventional methods for tumor organoid culture, including matrix-based methods, hanging drop, spinner or rotating flask, and nonadhesive surface, primarily generate multicellular aggregates containing single or multiple cell types 113. These tumor organoids are often simple, disorganized, and lack tissue-level characteristics. In contrast, microfabrication techniques, such as 3D bioprinting and organ-on-a-chip, can generate more advanced tumor organoids with spatial structures and containing mechanical/biochemical cues of TME 114. In addition, genetic engineering can program cell behaviors at genomic levels and allows the investigation of interactions between nontransformed and transformed cells. These methods for tumor organoid culture were summarized and compared in **Table 2**.

## Applications of tumor organoids

By effectively recapitulating tumor heterogeneity of cancer patients, tumor organoids have been extensively applied to cancer modeling, basic cancer research, and cancer therapy.

### Cancer modeling

Drug sensitivity in patient-derived samples is detected generally through two approaches, namely short-term cultivation of tumor sections and xenotransplantation of tumor tissues into immunodeficient mice 115,116. Short-term culture can be used for *in vitro* screening on an appropriately large scale but is limited by the low proliferative capacity of tumor sections. Xenotransplantation can be used for *in vivo* screening but is resource- and labor-intensive due to the need for many mice.

To dissolve the dilemma, living tumor organoid biobanks were established, which allowed *in vitro* high-throughput drug screening in patient-derived samples (**Figure 1C**). Wetering *et al.* constructed a tumor organoid biobank from 20 consecutive CRC patients, which closely recapitulated crucial features of the original tumors 19. Moreover, the “living biobank” showed a spectrum of genetic changes that corresponded well with prior large-scale mutational analyses of CRCs. Similarly, Meister *et al.* generated a biobank comprising 19 pediatric RMS tumor organoids 30. Molecular, histological, and genetic characterization showed that the models closely resembled the parental tumors, with genetic stability up to 6 months. Moreover, the models could be engineered by CRISPR/Cas9 with TP53 knockout in an embryonal RMS model, leading to replicative stress drug sensitivity. Therefore, tumors of mesenchymal origin can be employed to produce an organoid biobank.

**Figure 6:**
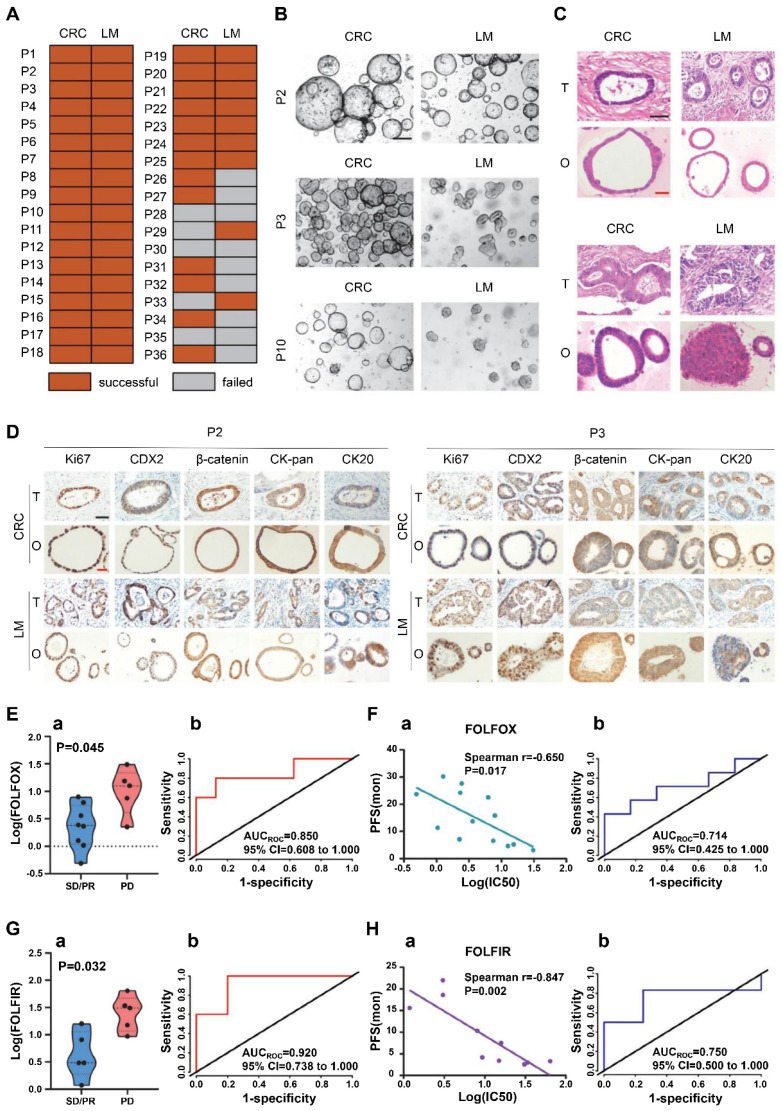
Patient-derived organoids from colorectal cancer (CRC) and paired liver metastasis (LM) predicted chemotherapeutic response. (A) Organoid culture success rate from CRC and LM tissues of patients with colorectal cancer liver metastasis (CRLM). (B) CRC and LM organoids from different CRLM patients showed three typical characteristics in the bright field. (C) H&E staining of CRC/LM organoids and corresponding parental tumors. T: parental tumors; O, CRC, or LM organoids. (D) Immunohistochemical staining of CRC/LM organoids and corresponding parental tumors with Ki-67, CDX2, β-catenin, CK-pan, and CK20. (O, CRC or LM organoids; T, parental tumors). (E) a, IC50 values of organoids for FOLFOX chemotherapy from SD/PR (n = 8) and PD patients (n = 5). b, An ROC curve showed the predictive efficacy of organoids for FOLFOX chemotherapy. (SD: stable disease; PR: partial response; PD: progressive disease). (F) a, Correlation between the IC50 values of organoids and progression‐free survival (PFS) for patients (n = 13). b, An ROC curve showed the predictive efficacy of organoids for the clinical prognosis of patients receiving FOLFOX treatment. (G) a, IC50 values of organoids for FOLFIRI chemotherapy from SD/PR (n = 5) and PD patients (n = 5). b, An ROC curve showed the predictive efficacy of organoids for FOLFIRI treatment response. (H) a, Correlation between IC50 values of organoids and PFS of patients (n = 10). b, An ROC curve showed the predictive efficacy of organoids for the clinical prognosis of patients receiving FOLFIRI treatment. Black scale bar, 200 µm; red scale bar, 100 µm. Adapted with permission from 118, copyright 2022, Wiley.

In addition, metastatic tumor organoid biobanks can also be established. Fujii* et al.* produced a 55 CRC organoid-containing biobank from various clinical stages and histological subtypes, including rare ones 117. The organoids recapitulated the differentiation capacity and histopathological grade of the original tumors. The paired primary and metastatic organoids had similar niche factor requirements and genetic profiles, and the metastasis-derived organoids showed higher metastatic capability than primary ones. The CRC organoid biobank provided insights into colorectal tumorigenesis and patient-centered treatment development. Shaobo Mo *et al.* constructed a CRC liver metastasis (CRLM) organoid biobank 118. Fifty organoids derived from CRC and paired LM tissues of 25 CRLM patients were successfully cultured (**Figure 6A**). Paired CRC and LM organoids had similar features but retained their heterogeneity. CRC and LM organoids exhibited three typical features (**Figure 6B**). P2 patient-derived CRC and LM organoids presented thin-walled cystic structures; P3 patient-derived LM organoids presented irregular solid structures, while CRC organoids showed thick-walled cystic structures; P10 patient-derived CRC organoids showed thin-walled cystic structures, while LM organoids exhibited solid spherical structures. Then, H&E staining showed that CRC and LM organoids recapitulated patient-derived heterogeneous morphology from thin-walled cystic to solid structures (**Figure 6C**). Moreover, immunohistochemical staining revealed that the expression pattern of crucial molecular markers, including Ki67, CDX2, β-catenin, CK-pan, and CK20 in CRC/LM organoids and original tumors, was entirely consistent (**Figure 6D**).

The tumor organoid capability of reflecting the chemotherapeutic response of CRLM patients was evaluated. Of 13 CRLM patients receiving FOLFOX chemotherapy, 5 were evaluated as progressive disease (PD) and 8 as stable disease (SD)/partial response (PR). Of 10 CRLM patients receiving FOLFIRI chemotherapy, 5 were assessed as PD and 5 as SD/PR 118. IC50 of FOLFOX or FOLFIRI significantly differed between organoids from SD/PR versus PD patients (**Figure 6E-a and G-a**). Moreover, CRC/LM organoid treatment data *in vitro* highly correlated with patients' clinical therapeutic response, with AUC values of 0.850 and 0.920 for FOLFOX and FOLFIRI therapies, respectively (**Figure 6E-b and G-b**). In addition, IC50 for both FOLFOX and FOLFIRI therapies *in vitro* correlated with progression-free survival (PFS) of the corresponding patients (**Figure 6F-a and 6H-a**). Meanwhile, ROCs based on FOLFOX and FOLFIRI combination treatments generated AUCs of 0.714 (**Figure 6F-b**) and 0.750 (**Figure 6H-b**), respectively. These findings suggest that the tumor organoid biobanks may predict the risk of disease progression for CRLM patients receiving FOLFOX or FOLFIRI combination chemotherapy.

Currently, researchers have established various types of human cancer organoid biobanks, such as GC 119, breast cancer 21, bladder cancer 120, childhood kidney cancer 121, glioblastoma 122, and neuroendocrine neoplasms 123. These biobanks have retained the genome landscapes of parental tumors and bridged fundamental cancer research and anticancer therapy.

### Basic cancer research

It is essential for fundamental cancer research to establish reliable preclinical models. However, tumorigenesis is a complex process, and enormous TME discrepancies between patients and current models are one of the restrictions that block progress in cancer research. Currently, tumor organoids are emerging as an efficient tool to explore the cellular and molecular processes in cancer progression (**Figure 1C**).

Zhang *et al.* constructed CRC organoids using a hanging drop method 124. They reported that IGF2, markedly upregulated in CAFs compared to normal fibroblasts, stimulated IGF1R, which is highly expressed on CRC cells. Moreover, YAP1 functioned as a pivotal downstream effector to mediate the oncogenic signaling of IGF2-IGF1R. Through the CRC organoids and *in vivo* studies, they found that co-targeting IGF1R and YAP1 with picropodophyllin and verteporfin (IGF1R and YAP1 inhibitors, respectively) showed higher antitumor effects than picropodophyllin monotherapy. In another study, to clarify the influence of inflammatory microenvironment on the CRC response to immune checkpoint inhibitors (ICIs), Qiaoqi Sui *et al.* constructed high microsatellite instability (MSI-H) CRC organoids in Matrigel 49. Local but not systemic immune response inhibition was confirmed in patients' co-cultures of paired organoid cells and T cells. Moreover, single-cell RNA sequencing revealed that neutrophil leukocytes were crucial players in immune suppression *via* CD80/CD86-CTLA4 signaling. Therefore, inflammatory conditions and an elevated neutrophil-to-lymphocyte ratio predict poor tumor response to ICIs in MSI-H CRCs.

Given that genomic drivers play a pivotal role in tumorigenesis, Lam and co-workers employed human liver organoids to recapitulate the early stages of human liver carcinogenesis from genetic lesions of TP53 loss and L3 loop R249S mutation 125. They found that CRISPR knockout of TP53 in liver organoids consistently translated to tumor-like morphological changes, increased stemness, and uncontrollable *in vitro* propagation. To mimic TP53 status in human HCC, they overexpressed mutant R249S in TP53 knockout organoids. A spontaneous enhancement in bona fide HCC histology and tumorigenic potentials were observed in xenotransplantations. Finally, they indicated distinct tumorigenic effects from TP53 loss and L3 mutations, which both endow normal hepatocytes with early clonal advantages and prosurvival functions.

### Anticancer therapy

#### Drug development and testing

Reliable tumor models are indispensable to discovering effective anticancer drugs. Although PDCs are extensively used to predict the sensitivity of anticancer drugs 126, these predictions correspond to a low success rate of clinical trials due to the incapability of recapitulating TME 127. PDXs are constructed by transplanting human tumor fragments into immunodeficient mice. However, the human matrix is gradually replaced by the mouse matrix and thus lacks an intact immune microenvironment, which is not qualified for immunomodulator detection 128. In contrast, tumor organoids make up the defects of both these models by effectively reproducing the original tumor tissue specificity and tumors' responses to drugs (**Figure 1C**).

Over the past decade, tumor organoids have been extensively applied to drug screenings. For example, Kopper *et al.* established a protocol to culture patient-derived tumor cells in basement membrane extract supplemented with a specific medium. This matrix enabled effective derivation and long-term expansion of ovarian cancer (OC) organoids 129. Through this protocol, they constructed 56 OC organoids from 32 patients, covering all main OC subtypes. OC organoids recapitulated genomic and histological characteristics of the parental tumors, indicating intra- and interpatient heterogeneity. As such, they could apply to drug-screening assays and detect different tumor subtype sensitivity to the gold standard platinum-based chemotherapy. Moreover, OC organoids could be xenografted for *in vivo* drug-sensitivity testing.

Driehuis *et al.* established 30 patient-derived organoids from pancreatic and distal bile duct tumors 20. The tumor organoids reproduced tumor histology and genetic alterations of pancreatic cancer. *In vitro* testing of 76 drugs revealed therapeutic responses not leveraged in the clinical presently. For example, protein arginine methyltransferase 5 (PRMT5) inhibitor EZP015556 effectively inhibited both methylthioadenosine phosphorylase (MTAP)- and MTAP+ tumor organoids, both of which were hallmarked by high 5'-methylthioadenosine (MTA) levels. Overall, the work provided a tumor organoid platform to find novel antitumor therapeutics.

In addition to primary tumor organoids, metastatic tumor organoids can be established for drug testing. Vlachogiannis *et al.* established a living biobank of tumor organoids from metastatic, heavily pretreated gastroesophageal and CRC patients recruited in phase 1/2 clinical trials 130. The tumor organoids recapitulated phenotypic and genotypic profiling of the parental tumors to a high degree. Then, 55 drugs now in phase I-III clinical trials or in clinical practice were tested using the organoids. Moreover, the responses of anticancer agents in organoids and organoid-based orthotopic xenograft mouse models were consistent with the patient's responses in clinical trials, suggesting the great potential of PDOs for drug screening.

High-throughput drug testing with organoid cultures is impractical due to the limited organoids available for each case and the vast cost and time needed for *in vitro* expansion. To this end, Jumpei Kondo *et al.* developed a cancer tissue-originated spheroid (CTOS) method for the high-throughput screening of 2427 drugs 131. They generated CTOSs from xenograft tumors and applied an automatic spheroid handler to select spheroids based on appearance and size (70-100 µm). CTOS passages in xenograft tumors induced negligible alterations of morphologies and genomic status and effectively expanded the production capacity of CTOSs. The panel of CRC CTOS lines showed different responses to the hit compounds, indicating the applicability of this system for personalized drug testing.

As an alternative to new drug development, drug repurposing is more cost-effective and time-saving. Srimongkol *et al.* constructed retinoblastoma (RB) organoids that recapitulated the original tumors' genomic features 132. Then, 133 FDA-approved drugs were tested in RB organoids, and candidate drugs were screened according to potency and cytotoxicity. Sunitinib was identified as a more effective inhibitor of tumor cell proliferation in RB organoids and showed lower toxicity to normal retinal organoids than melphalan or topotecan. These results suggest that sunitinib could be repurposed for RB chemotherapy.

#### Personalized medicine

Since the patient-derived tumor organoids preserve the features of the original tumors, they can provide the basis for formulating personalized clinical anticancer regimens. Applications of tumor organoids in personalized chemotherapy, radiotherapy, and immunotherapy are introduced as follows (**Figure 1C**).

##### Chemotherapy

Chemotherapy is one of the most effective modalities for cancer therapy. However, the responses of different patients to the same chemotherapeutic agents are often distinct. For personalized medicine, tumor organoids are used as an adjuvant tool to capture the direct impact of chemotherapeutics on tumors and identify the available treatment decisions for patients.

Wang *et al.* established 212 LCOs from 107 patients, mainly from malignant serous effusions 133. Drug sensitivity tests for chemotherapy and targeted therapy (e.g., nab-paclitaxel and osimertinib) were performed in the LCOs to predict clinical responses to respective treatments. Finally, the LCOs accurately predict the clinical responses to various treatments in this cohort of patients with advanced lung cancer.

**Figure 7:**
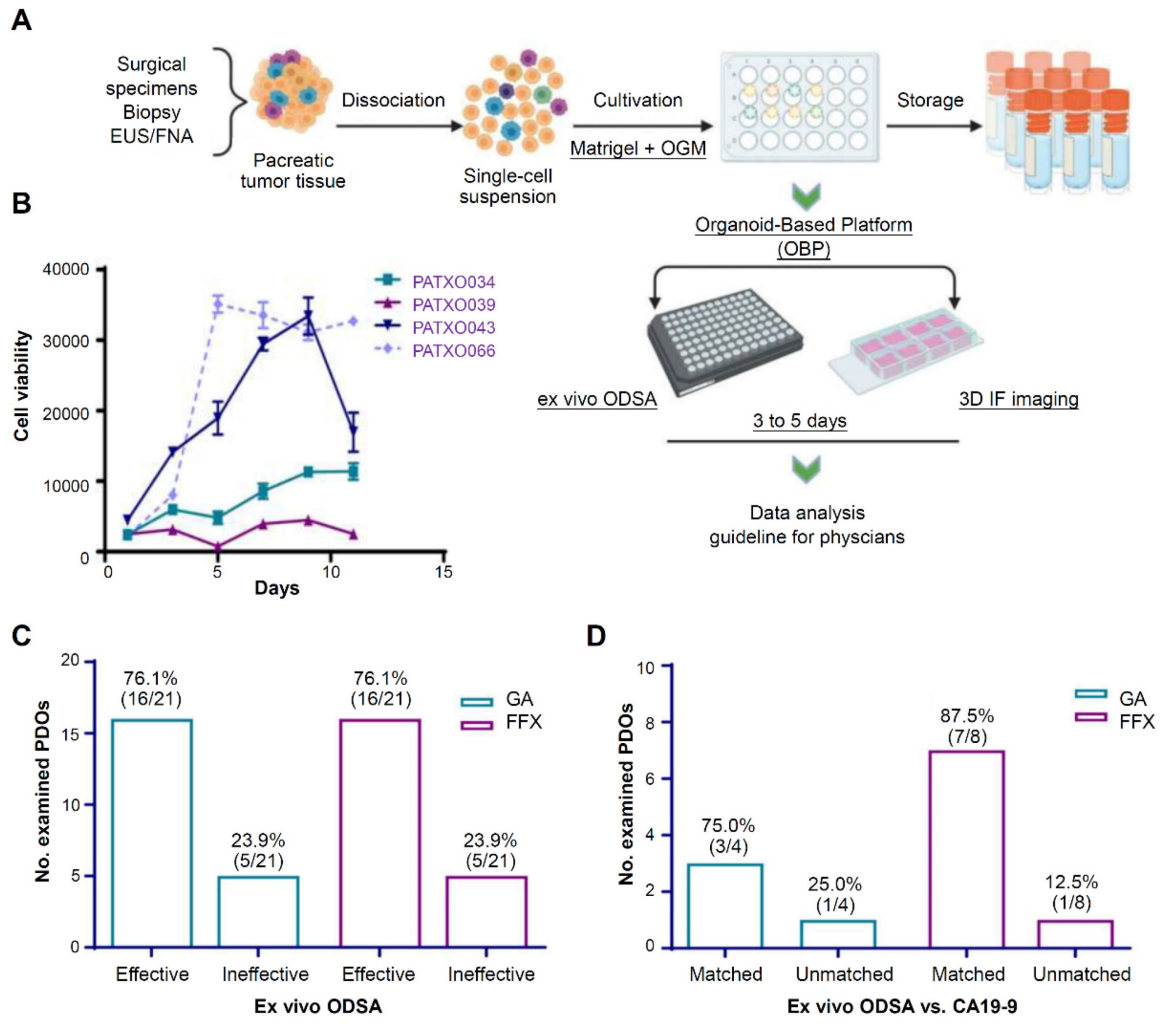
Tumor organoid-guided personalized treatment in pancreatic ductal adenocarcinoma (PDAC). (A) Procedures for the establishment of PDAC organoid-based platform. (B) Growth curves for 4 PDAC patient-derived tumor organoids. (C) Summary of PDO response to GA or FFX, as measured by the *ex vivo* ODSA at elevated drug doses. (D) Consistency of the *ex vivo* tumor organoid responses with carbohydrate antigen 19-9 (CA19-9) status in the corresponding tumor patients (“matched” indicates consistent findings). GA: gemcitabine plus nab-paclitaxel; FFX: 5-fluorouracil, irinotecan and oxaliplatin. Adapted with permission from 134, copyright 2022, Amer Soc Clinical Investigation Inc.

Currently, 2 U.S. FDA-approved chemotherapy regimens mainly apply to PDAC treatment: gemcitabine plus nab-paclitaxel and the combination of 5-fluorouracil, irinotecan, and oxaliplatin (FFX) 134. Since there is a lack of reliable methods to predict patient responses, patients are often switched from one regimen to another due to poor clinical efficacy. Kang *et al.* established an organoid platform to visually detect drug efficacy and associated tumor-stroma modulation (**Figure 7A**) 134. Growth curves from 4 different PDOs showed different profiles over 11 days, indicating the heterogeneity of PDAC organoid growth (**Figure 7B**). At elevated drug doses, response rates of PDOs to gemcitabine plus nab-paclitaxel and FFX were 76.1% (**Figure 7C**). Then, they compared the ODSA-measured effectiveness of each regimen with the effectiveness of each regimen in PDAC patients, as measured by decreases in serum CA19-9 levels (**Figure 7D**). A high consistency of effectiveness was observed between the PDOs and the PDAC patients for gemcitabine plus nab-paclitaxel or FFX. Therefore, the organoid-based platform helps to select personalized therapies for PDAC patients.

Most patients with biliary tract cancer (BTC) are diagnosed at an unresectable stage, for whom systemic chemotherapy remains the mainstay of palliative treatment 135. However, chemotherapeutic efficacy is highly variable in BTC patients. Xiaoxue Ren *et al.* established 61 tumor organoids from 82 BTC patients that recapitulated the genetic and histological characteristics of the parental tumors 136. These PDOs exhibited different sensitivity to the chemotherapeutic agents, such as gemcitabine, 5-fluorouracil, cisplatin, and oxaliplatin. The drug effectiveness results from the PDOs were further confirmed in the 92.3% (12/13) patients. Furthermore, they identified the correlation of gene expression of BTC PDOs with drug sensitivity, facilitating the prediction of chemotherapy responses in BTC patients and selecting suitable chemotherapeutics for individual BTC patients.

The administration of systemic chemotherapy before resection (i.e., neoadjuvant chemotherapy) has been extensively accepted as an option for cancer patients 137. Adding irinotecan to the chemotherapy regimen increases the pathologic complete response of patients with advanced rectal cancer but brings additional toxicities. Tao Lv *et al.* established tumor organoids from rectal cancer patients, which were treated with irinotecan or 5-fluorouracil for 6 days 138. Irradiation was conducted synchronously at a dose of 8Gy. Organoid dimensions were monitored every 3 days for 24 days. They found that irinotecan sensitivity of tumor organoids effectively predicts rectal cancer patients' clinical responses toward irinotecan.

Notably, as the period during which patients can receive neoadjuvant chemotherapy is short, an optimal drug regimen must be identified as soon as possible 139. Lyudmyla Demyan *et al.* collected 136 samples from 117 PDAC patients, including fine needle aspiration/biopsy and surgical resections 140. These PDO responses to chemotherapy were well correlated with pathological response to neoadjuvant chemotherapy, particularly oxaliplatin. In particular, drug screening could be rapidly performed in the organoids with data generated within 7 days of tissue resection, indicating a clinically relevant timeline.

##### Radiotherapy

Cancer patients' responses to radiotherapy are highly heterogeneous and difficult to identify before surgery. Kuo-Shun Hsu *et al.* generated organoids from normal human intestines and CRC of neoadjuvant therapy patients 141. The adenomas-derived organoids containing a logarithmically-expanded Lgr5^+^-intestinal stem cell population retained the radioresistant property of normal colorectal organoids. In contrast, organoids derived from malignant transformation of patients showed prominent radiosensitivity because of decreased homologous recombination-mediated DNA repair. Consistently, clinical trials revealed that CRC patient responses to neoadjuvant chemoradiation correlated closely with their organoid D0 values. Overall, organoid radiation responses could predict extensive radiation sensitivity precisely that occurred in CRC patients.

In addition, Karuna Ganesh *et al.* cultured 65 rectal cancer organoids from patients with primary, metastatic, or recurrent diseases 142. These organoids recapitulated molecular characteristics of the parental tumors, and their *ex vivo* responses to clinically relevant chemoradiation treatments were closely correlated with the clinical efficacy of individual patients. Furthermore, upon inoculation into murine rectal mucosa, human rectal cancer organoids showed different responses to chemotherapy, as observed clinically. Therefore, the *ex vivo* rectal cancer organoid platform combined with *in vivo* endoluminal propagation in animals can effectively predict chemoradiation sensitivity of patients with rectal cancer. Similarly, Ye Yao *et al.* established a tumor organoid biobank from patients with locally advanced rectal cancer recruited in a phase Ⅲ clinical trial 57. The organoids closely retained the pathophysiology and genetic features of the original tumors. Moreover, the organoid responses were highly consistent with chemoradiation responses in patients, with 91.97% specificity, 84.43% accuracy, and 78.01% sensitivity, indicating that the organoids were an effective adjuvant diagnostic tool in rectal cancer treatment.

Cervical cancer is a primary health issue in developing countries. Hua Huang *et al.* constructed PDOs from 67 patients containing heterogeneous cervical cancer, which closely recapitulated the genomic and histopathological features of parental tumors 48. The *in vitro* sensitivity of PDOs effectively predicted the heterogenic radiotherapy efficacy of the patients. In addition, they co-cultured the PDOs with paired tumor-infiltrating lymphocytes, which showed clear responses corresponding to established immunotherapy efficiency markers, providing guide therapy in prospective interventional cervical cancer trials.

##### Immunotherapy

The tumor immune process includes several stages, i.e., tumor antigen release, antigen presentation, activation and proliferation of effector T cells, migration and infiltration of T cells into tumor tissues, and recognition and removal of tumor cells by T cells 143. Any abnormality in these stages will lead to immune failure that the immune system is suppressed to fail to kill tumor cells. Therefore, tumor immunotherapy predominantly involves restoring the patients' antitumor immune response 143,144. Immune checkpoint therapy, which improves antitumor immune responses by targeting regulatory pathways in T cells, has obtained critical clinical advances. The U.S. FDA has approved three ICIs (i.e., nivolumab, pembrolizumab, and ipilimumab) for melanoma treatment 145. Immune checkpoint therapy has joined the ranks of radiation, chemotherapy, surgery, and targeted therapy as a pillar of cancer treatment.

Despite promising prospects, tumor heterogeneity makes selecting cancer patients for ICI treatment difficult. Considering the ability of tumor organoids to recapitulate tumor heterogeneity, Giosue Scognamiglio *et al.* constructed tumor organoids from patients with chordoma by dissociating the tumor tissues and seeding the obtained single cells in Matrigel 146. Organoids derived from PD-L1-positive patients contained both tumor cells and PD-1/CD8-positive lymphocytes and showed a prominent dose-dependent dimension decrease of 50% in response to nivolumab treatment. Meanwhile, treatment with nivolumab promoted cell death in both PD-L1-positive and negative organoids. These results indicated that organoids may be a helpful tool to predict individual sensitivity to immunotherapy.

The diffuse-type gastric cancer (DGC) is a GC subtype with low HER2 expression and poor sensitivity to ICIs. Long Qin *et al.* developed an anti-uPAR monoclonal antibody that hindered the coupling of urokinase-plasminogen activator to uPAR 147. Combining anti-uPAR and anti-PD-1 markedly suppressed tumor growth and prolonged survivals in the cell line- and patient-derived xenograft mouse models. More importantly, uPAR chimeric antigen receptor-expressing T cells killed DGC PDOs effectively and prolonged the survival of the established mouse models, especially when combined with PD-1 inhibitors. This study provided a new regimen for DGC immunotherapy.

MSCs accumulate in primary tumors to promote tumor growth and recruit immune cells into TME. To predict the immunotherapy efficacy of HCC patients, Zhengyu Zou *et al.* established an HCC organoid-on-a-chip model where peripheral blood mononuclear cells (PBMCs) and HCC PDOs were co-cultured with CAFs and MSCs 50. A multilayer microfluidic chip is fabricated for high-throughput co-culture. CAF-PDO-PBMC and MSC-PDO-PBMC models could predict the patient's responses to anti-PD-L1 drugs more precisely than conventional PDOs. In particular, they shortened the culture time to 1-2 weeks, providing the clinical application possibility of personalized immunotherapy.

A lack of preclinical models recapitulating neoantigen features of parental tumors limits the development of neoantigen-directed immunotherapies. To screen neoantigens, Wenwen Wang *et al.* constructed patient-derived hepatobiliary tumor organoids 148. The comparisons between 9203 predicted neoantigen-peptides from 2449 mutations of tumor tissues and 9991 peptides from paired organoid mutations indicated that organoids retained human leukocyte antigen alleles, genetic features, and neoantigen landscape of original tumors. Nine immunogenic peptides were validated and shared by at least two individuals. The antitumor capability of the validated immunogenic peptide-reactive CD8 cells was confirmed through the organoid-killing assay, which was further improved when combined with ICIs. This study exemplified that tumor organoids could be applied to identify neoantigen-peptides in personalized immunotherapy.

## Conclusions and future perspectives

Tumor organoids, an *in vitro* tumor model, can recapitulate tumor heterogeneity effectively and fill the gaps between PDCs and PDXs. Tumor organoids have been extensively applied to cancer modeling, basic cancer research, and anticancer therapy. Despite promising prospects, there is still tremendous room for improvement.

One crucial hurdle is the low culture success rate. The success rate of organoid derivation and reliable *in vitro* expansion is < 30% in many cancer subtypes 149. Culture success mainly depends on the available starting material, biopsy and resection, and tumor cellularity, which vary significantly between tumor types 150. Ooft, S. N. *et al.* established 31 organoids from 54 CRC patients with a success rate of 57.4% 151. By optimizing the culture medium components, Sachs *et al.* enhanced the success rate of breast cancer organoid cultivation to > 80% 21. However, it remains unclear to what degree the success rate can be enhanced for other cancer types and if the success rate can meet the need for clinical use. In addition, most tumor organoids established thus far are derived from epithelial tumors and a small portion from nonepithelial tumors, such as osteosarcoma and RMS 30,152. Therefore, more efforts are needed to improve the success rate of nonepithelial tumor organoid culture.

Another unavoidable problem is batch-to-batch variability due to non-standardized cancer tissue samples and subsequent processing, non-specific and ill-defined medium formulations, and heterogeneous, animal-derived matrices. For example, animal-derived serum in the organoid culture medium leads to a non-standard culture platform. Specifically, fetal bovine serum (FBS) derived from the fetal calf blood contains massive peptides, proteins, lipids, hormones, carbohydrates, and small-molecule nutrients 149. Animal origin and seasonal and geographic differences in serum harvest bring variability in soluble-component concentrations across suppliers and batches. In addition, Matrigel, the most common matrix for cancer organoid cultivation over the past decade, is mouse-derived and thus shows considerable batch-to-batch variability. Moreover, Matrigel contains indefinite and xenogenic impurities that potentially affect organoid phenotype 153. Even after special processing, growth-factor-reduced Matrigel exhibits merely ~53% batch-to-batch consistency in protein content 154.

To this end, engineered culture medium components and matrices have been developed. For example, Willert *et al.* established a coexpression system comprising mammalian cells transfected with afamin- and Wnt-encoding vectors, generating a Wnt-conditioned medium to replace FBS for cultivating several cancer organoids 155. However, FBS is still included during initial mammalian cell propagation before the harvest of the conditioned medium, taking the risk of contaminating the medium with FBS-derived components. In addition, to achieve batch-to-batch consistency, synthetic materials, such as HA, PEG, and gelatin, have been extensively developed as engineered matrices for organoid cultivation, as mentioned above 156-159. However, engineered matrices often exhibit lower culture efficiency than Matrigel, and synthetic matrices that enable the organoid culture of one species or tissue are generally not directly transferred to others, restricting their extensive application. Future advancements in material engineering and polymer science must address these limitations.

Organoid cultivation needs a specific culture medium containing massive, costly growth factors. Aside from the high cost, the complexity of the culture process makes it difficult to achieve large-scale organoid culture. Although simplified organoid models contribute to their large-scale generation, oversimplification may compromise their ability to recapitulate tumor features. Therefore, the balance between minimizing the complexity of culture procedures and maximizing the resemblance to parental tumors should be considered when designing engineering approaches 160,161.

A lack of multi-cellular components and vasculature is still considered a significant defect of tumor organoids. Primary tumor organoids contain stromal cells, such as immune and fibroblast cells; however, they gradually lose these cells during the long-term culture 162. 3D co-culture, decellularization techniques, and microfluidic devices have been developed to mimic *in vivo* cancer-stromal cell interplay 163. However, most co-culture systems use stromal cell lines or mouse-derived primary stromal cells, and the clinical relevance is questionable. The future direction for the co-culture system is using matched patient-derived stromal cells to study cancer cell-stroma interactions in patients. In addition, current tumor organoids generally have a limited size due to the lack of vasculature restricting nutrient absorption. As mentioned above, bioprinting and organ-on-a-chip techniques have been explored to develop vascularized organoids. However, these organoid models can still not mimic crosstalk between tumors and other tissues completely, thus failing to reflect the developmental and pathological features of their *in vivo* parental tumors. Research efforts to address these limitations are still needed urgently.

With the popularity of 3D bioprinting in tumor organoid construction, biomaterials with precisely tunable properties are in great demand 164. In this respect, many researchers set out to optimize commonly used bioinks and develop new materials with organ-specific properties to meet the requirements of mechanical strength and cytocompatibility for 3D bioprinting 165-167. In addition, 3D bioprinting currently produces only small organ and tissue models. Producing large tissues of clinically relevant shapes and sizes remains challenging due to the heterogeneity and architecture complexities of native tissues. Moreover, generating large constructs needs prolonged printing for existing production capabilities. Techniques to speed up the printing process are urgently needed. Another limitation is that most bioprinters are incompatible with available bioinks, or cannot print multiple bioinks simultaneously. Therefore, synchronous developments of bioprinters and biomaterials/bioinks are necessary for large-scale bioprinting of 3D tissues.

Lack of control over mechanical and biochemical signals is another primary limitation of tumor organoids. Adding spatiotemporal mechanical/chemical gradients in the ECM will advance *ex vivo* tumor organoids toward *in vivo* tumors. For example, as mentioned above, Fang *et al.* successfully simulated the human intestine peristalsis in a CRC organoid by designing a microdevice on a microfluidic chip 91. They found that peristalsis prevented cellular uptake of ellipticine-loaded micelles, underlining the significance of mimicking mechanical/chemical signals.

Organ-on-a-chip comprising microchannels and complex chambers can achieve dynamic fluidic circulation and interconnect to mimic multiorgan communication. In particular, organ-on-a-chip can manipulate biochemical/physical cues of TME, such as pH gradient, oxygen levels, molecular gradient, nutrient diffusion, and, most importantly, the flow of circulating cell components. However, traditional manufacturing techniques for chip devices are labor-intensive, time-consuming, and unable to deposit living components precisely. Therefore, prototyping bioprinting organ-on-a-chip is becoming a research hotspot to resolve these obstacles. Organ-on-a-chip and bioprinting can be merged through post-integration and single-step production strategies. The post-integration strategy fully uses microfluidic processing to construct chip containers with complicated microflow channels 168,169. Then, the bioprinting process introduces biological components into these containers. For a single-step production strategy, the assembly of chip containers and biological composition can be implemented simultaneously by 3D printing 170. The single-step strategy facilitates the development of a customized tumor-on-a-chip model through versatile architecture design. However, a single-step strategy requires the shell material to be biocompatible, printable, and robust enough for long-term culture, restricting the selection of shell materials. The prolonged printing process in the single-step assembly may reduce the viability of living cells. Moreover, printers meeting the complicated printing process are hardly commercially available. More efforts should be made to address these obstacles in a single-step strategy, including material selection, equipment accessibility, and process time, for large-scale production and clinical translation.

Despite facing many challenges, tumor organoid development has been booming. Tumor organoids are compatible with various therapeutics, including proteins, small molecules, and cell-based drugs. They can also produce diverse readouts, such as biomarker secretion, cytotoxicity, and infiltration of immune cells. The 3D tumor organoid models apply to screening various therapies in the solid tumor setting and giving accurate insight into their efficacy. Inspiringly, in August 2022, the U.S. FDA approved the first new drug for clinical trials (NCT04658472) entirely based on the research data from tumor organoids. Furthermore, in January 2023, the U.S. FDA declared that animal tests are no longer required before human drug trials, signifying that tumor organoids are gradually accepted and would play a more and more critical role in cancer research and therapy 171.

## Figures and Tables

**Table 1:** Summary of various cellular materials additionally supplemented for tumor organoid co-culture.

Cell materials	Tumor types	Co-culture methods	Molar ratio (organoids/tumor cells: additional cellular materials)	Functions	Ref
CAFs	PDAC	Digest organoids into single cells or small aggregates and mix them with patient-derived CAFs	1:1	Study the stroma‑mediated chemoresistance	37
	CRC	Digest organoids to single-cell suspensions and mix them with the patient-matched normal fibroblasts and CAFs	1:5	Investigate the tumor cells-CAF interactions	36
	CRC	The patient-derived CAFs are added to the CRC organoids that have been cultured for 2 days	2~3:1	Evaluate standard-of-care drugs of CRC	34
Immune cells	PDAC	Macrophages and cancer cells share a culture medium but are separated into two spaces of transwell without direct cell-cell contact	3:1	Investigate the macrophage-associated gemcitabine resistance	44
	PDAC	CD3/CD28 antibodies pre-activated, OVA-specific T cells are mixed with PDAC organoids that have been cultured for 5 days	1:1000~2500	Recapitulate T cell-infiltrated TME for drug testing	42
	Cervical cancer	Expand tumor-infiltrating lymphocytes *ex vivo* and co-culture them with paired cervical cancer organoids	NA	Model individual responses to adoptive T-cell therapy	48
	CRC	Co-culture tumor organoids with paired tumor-infiltrating lymphocytes or PBMC-derived T cells	1:20	Study the influence of inflammatory conditions on tumor sensitivity to immune checkpoint inhibitors	49
	Non-small cell lung cancer, CRC	Co-culture tumor organoids with autologous PBMCs for 2 weeks	1:20	Evaluate T-cell-based immunotherapy *ex vivo* for the individual patient	41
	HCC	Co-culture patient-derived tumor cells with autologous PBMCs and allogenic mesenchymal stromal cells.	10:30:1	Precisely assess the patients' responses to anti-PD-L1 drugs	50
ECs	HCC	Co-culture PDX-derived cells with HUVECs	1.5:2	Understand and target the interactions between the immune milieu and angiogenesis	45
ECs and MCs	PDAC	Co-culture patient-derived tumor cells with human iPSC-derived ECs and MCs	Tumor cells: ECs: MCs = 12:8.4:24	Explore the drug resistance and recurrence of PDAC	38
	HCC	Co-culture patient-derived tumor cells with human iPSC-derived ECs and MCs	Tumor cells: ECs: MCs = 10:2:2	Investigate the effects of TME on HCC development	46
ECs and immune cells	PDAC	Co-culture patient-derived tumor cells with paired PBMCs and HUVECs	Tumor cells: HUVECs: PBMCs = 1:2:2	Investigate the role of Jagged1 in PDAC development	47

CAFs: cancer-associated fibroblasts; PDAC: pancreatic ductal adenocarcinoma; CRC: colorectal cancer; PBMCs: peripheral blood mononuclear cells; ECs: endothelial cells; HCC: hepatocellular carcinoma; HUVECs: human umbilical vein endothelial cells; MCs: mesenchymal cells; iPSCs: induced pluripotent stem cells; TME: tumor microenvironment; NA: not available.

**Table 2:** Advantages and disadvantages of various methods for tumor organoid construction.

	Matrix-based methods	Hanging drop	Spinner or rotating flask	Nonadhesive surface	Organ-on-a-chip	3D bioprinting	Genetic engineering
Advantages	1) Provide ECM scaffolds and simulate interstitial tissue 2) Promote cell adhesion and growth	1) Maintain 3D tissue architecture 2) Require a tiny amount of medium 3) Facilitate efficient gas exchange 4) Can adjust organoid dimensions	1) Simple operation 2) Facilitate the exchange of nutrients and waste 3) Can adjust organoid dimensions	1) Simple operation 2) Strong cell-cell interaction	1) Can study tumor multiorgan metastasis and tumor-stroma interactions 2) Engineer perfused vascular networks 3) Reproduce mechanical/biochemical cues of TME 4) Can control the dimensions and shapes of organoids 5) Cost less time for organoid generation	1) Can control the dimensions and shapes of organoids 2) Engineer perfused vascular networks 3) Can customize various TME features	1) Can investigate interactions between nontransformed and transformed cells 2) Can simultaneously analyze antitumor activity and accompanied toxicity
Disadvantages	1) Batch-to-batch variability 2) Simple and disorganized, with limited tissue-level characteristics	1) Labor-intensive 2) Lack of cell-matrix interactions 3) Medium evaporation-caused osmotic pressure improvement affects cell growth	1) High consumption of culture medium 2) Sheer force inhibits cell growth	1) Difficult to control the organoid dimensions 2) Difficult to track the growth of each organoid	1) Labor-intensive and time-consuming fabrication process 2) Unable to deposit living components precisely	1) Lack of suitable bioinks and printers 2) Produce only small organ and tissue models	1) Lack of vasculature 2) Hinge on the disadvantages of derived normal organoids

ECM: extracellular matrix; TME: tumor microenvironment.

## References

[1] Siegel RL, Miller KD, Wagle NS, Jemal A (2023). Cancer statistics, 2023. CA Cancer J Clin.

[2] Lee J, Liu Z, Sa JK, Shin S, Wang J, Bordyuh M (2018). Pharmacogenomic landscape of patient-derived tumor cells informs precision oncology therapy. Nat Genet.

[3] Burrell RA, McGranahan N, Bartek J, Swanton C (2013). The causes and consequences of genetic heterogeneity in cancer evolution. Nature.

[4] McMillin DW, Negri JM, Mitsiades CS (2013). The role of tumour-stromal interactions in modifying drug response: challenges and opportunities. Nat Rev Drug Discov.

[5] Zeigerer A, Wuttke A, Marsico G, Seifert S, Kalaidzidis Y, Zerial M (2017). Functional properties of hepatocytes in vitro are correlated with cell polarity maintenance. Exp Cell Res.

[6] Yoshida GJ (2020). Applications of patient-derived tumor xenograft models and tumor organoids. J Hematol Oncol.

[7] Julien S, Merino-Trigo A, Lacroix L, Pocard M, Goéré D, Mariani P (2012). Characterization of a Large Panel of Patient-Derived Tumor Xenografts Representing the Clinical Heterogeneity of Human Colorectal Cancer. Clin Cancer Res.

[8] Peng S, Creighton CJ, Zhang Y, Sen B, Mazumdar T, Myers JN (2013). Tumor grafts derived from patients with head and neck squamous carcinoma authentically maintain the molecular and histologic characteristics of human cancers. J Transl Med.

[9] Gould SE, Junttila MR, de Sauvage FJ (2015). Translational value of mouse models in oncology drug development. Nat Med.

[10] Xu H, Jiao D, Liu A, Wu K (2022). Tumor organoids: applications in cancer modeling and potentials in precision medicine. J Hematol Oncol.

[11] Sato T, Vries RG, Snippert HJ, van de Wetering M, Barker N, Stange DE (2009). Single Lgr5 stem cells build crypt-villus structures in vitro without a mesenchymal niche. Nature.

[12] Sato T, Stange DE, Ferrante M, Vries RG, Van Es JH, Van den Brink S (2011). Long-term expansion of epithelial organoids from human colon, adenoma, adenocarcinoma, and Barrett's epithelium. Gastroenterology.

[13] Jager M, Blokzijl F, Sasselli V, Boymans S, Janssen R, Besselink N (2018). Measuring mutation accumulation in single human adult stem cells by whole-genome sequencing of organoid cultures. Nat Protoc.

[14] Huang L, Holtzinger A, Jagan I, BeGora M, Lohse I, Ngai N (2015). Ductal pancreatic cancer modeling and drug screening using human pluripotent stem cell- and patient-derived tumor organoids. Nat Med.

[15] Steinbichler TB, Dudas J, Skvortsov S, Ganswindt U, Riechelmann H, Skvortsova II (2018). Therapy resistance mediated by cancer stem cells. Semin Cancer Biol.

[16] Dye BR, Hill DR, Ferguson MA, Tsai YH, Nagy MS, Dyal R (2015). In vitro generation of human pluripotent stem cell derived lung organoids. Elife.

[17] Driehuis E, Kretzschmar K, Clevers H (2020). Establishment of patient-derived cancer organoids for drug-screening applications. Nat Protoc.

[18] Nanki K, Toshimitsu K, Takano A, Fujii M, Shimokawa M, Ohta Y (2018). Divergent Routes toward Wnt and R-spondin Niche Independency during Human Gastric Carcinogenesis. Cell.

[19] van de Wetering M, Francies HE, Francis JM, Bounova G, Iorio F, Pronk A (2015). Prospective derivation of a living organoid biobank of colorectal cancer patients. Cell.

[20] Driehuis E, van Hoeck A, Moore K, Kolders S, Francies HE, Gulersonmez MC (2019). Pancreatic cancer organoids recapitulate disease and allow personalized drug screening. Proc Natl Acad Sci U S A.

[21] Sachs N, de Ligt J, Kopper O, Gogola E, Bounova G, Weeber F (2018). A Living Biobank of Breast Cancer Organoids Captures Disease Heterogeneity. Cell.

[22] Broutier L, Mastrogiovanni G, Verstegen MM, Francies HE, Gavarro LM, Bradshaw CR (2017). Human primary liver cancer-derived organoid cultures for disease modeling and drug screening. Nat Med.

[23] Tung YC, Hsiao AY, Allen SG, Torisawa YS, Ho M, Takayama S (2011). High-throughput 3D spheroid culture and drug testing using a 384 hanging drop array. Analyst.

[24] Lee GY, Kenny PA, Lee EH, Bissell MJ (2007). Three-dimensional culture models of normal and malignant breast epithelial cells. Nat Methods.

[25] Mosquera MJ, Kim S, Bareja R, Fang Z, Cai S, Pan H (2022). Extracellular Matrix in Synthetic Hydrogel-Based Prostate Cancer Organoids Regulate Therapeutic Response to EZH2 and DRD2 Inhibitors. Adv Mater.

[26] Eder T, Eder IE (2017). 3D Hanging Drop Culture to Establish Prostate Cancer Organoids. Methods Mol Biol.

[27] Djomehri SI, Burman B, Gonzalez ME, Takayama S, Kleer CG (2019). A reproducible scaffold-free 3D organoid model to study neoplastic progression in breast cancer. J Cell Commun Signal.

[28] Kleuskens M, Crispim JF, van Donkelaar CC, Janssen R, Ito K (2022). Evaluating Initial Integration of Cell-Based Chondrogenic Constructs in Human Osteochondral Explants. Tissue Eng Part C Methods.

[29] Kim M, Mun H, Sung CO, Cho EJ, Jeon HJ, Chun SM (2019). Patient-derived lung cancer organoids as in vitro cancer models for therapeutic screening. Nat Commun.

[30] Meister MT, Groot KM, de Souza T, Breunis WB, Frazer-Mendelewska E, Brok M (2022). Mesenchymal tumor organoid models recapitulate rhabdomyosarcoma subtypes. EMBO Mol Med.

[31] Tuveson D, Clevers H (2019). Cancer modeling meets human organoid technology. Science.

[32] Anderson NM, Simon MC (2020). The tumor microenvironment. Curr Biol.

[33] Yuan J, Li X, Yu S (2023). Cancer organoid co-culture model system: Novel approach to guide precision medicine. Front Immunol.

[34] Luo X, Fong E, Zhu C, Lin Q, Xiong M, Li A (2021). Hydrogel-based colorectal cancer organoid co-culture models. Acta Biomater.

[35] Spaeth EL, Dembinski JL, Sasser AK, Watson K, Klopp A, Hall B (2009). Mesenchymal stem cell transition to tumor-associated fibroblasts contributes to fibrovascular network expansion and tumor progression. Plos One.

[36] Dang H, Harryvan TJ, Liao CY, Danen E, Spalburg V, Kielbasa SM (2023). Cancer-Associated Fibroblasts Are Key Determinants of Cancer Cell Invasion in the Earliest Stage of Colorectal Cancer. Cell Mol Gastroenterol Hepatol.

[37] Schuth S, Le Blanc S, Krieger TG, Jabs J, Schenk M, Giese NA (2022). Patient-specific modeling of stroma-mediated chemoresistance of pancreatic cancer using a three-dimensional organoid-fibroblast co-culture system. J Exp Clin Cancer Res.

[38] Takeuchi K, Tabe S, Takahashi K, Aoshima K, Matsuo M, Ueno Y (2023). Incorporation of human iPSC-derived stromal cells creates a pancreatic cancer organoid with heterogeneous cancer-associated fibroblasts. Cell Rep.

[39] Paijens ST, Vledder A, de Bruyn M, Nijman HW (2021). Tumor-infiltrating lymphocytes in the immunotherapy era. Cell Mol Immunol.

[40] Neal JT, Li X, Zhu J, Giangarra V, Grzeskowiak CL, Ju J (2018). Organoid Modeling of the Tumor Immune Microenvironment. Cell.

[41] Cattaneo CM, Dijkstra KK, Fanchi LF, Kelderman S, Kaing S, van Rooij N (2020). Tumor organoid-T-cell coculture systems. Nat Protoc.

[42] Zhou Z, Van der Jeught K, Li Y, Sharma S, Yu T, Moulana I (2023). A T Cell-Engaging Tumor Organoid Platform for Pancreatic Cancer Immunotherapy. Adv Sci (Weinh).

[43] Xu C, Sui S, Shang Y, Yu Z, Han J, Zhang G (2020). The landscape of immune cell infiltration and its clinical implications of pancreatic ductal adenocarcinoma. J Adv Res.

[44] Jiang S, Deng T, Cheng H, Liu W, Shi D, Yuan J (2023). Macrophage-organoid co-culture model for identifying treatment strategies against macrophage-related gemcitabine resistance. J Exp Clin Cancer Res.

[45] Lim JTC, Kwang LG, Ho NCW, Toh CCM, Too NSH, Hooi L (2022). Hepatocellular carcinoma organoid co-cultures mimic angiocrine crosstalk to generate inflammatory tumor microenvironment. Biomaterials.

[46] Qiu R, Murata S, Cheng C, Mori A, Nie Y, Mikami S (2021). A Novel Orthotopic Liver Cancer Model for Creating a Human-like Tumor Microenvironment. Cancers (Basel).

[47] Choi J, Rim JH, Jang SI, Park JS, Park H, Cho JH (2022). The role of Jagged1 as a dynamic switch of cancer cell plasticity in PDAC assembloids. Theranostics.

[48] Huang H, Pan Y, Huang J, Zhang C, Liao Y, Du Q (2023). Patient-derived organoids as personalized avatars and a potential immunotherapy model in cervical cancer. iScience.

[49] Sui Q, Zhang X, Chen C, Tang J, Yu J, Li W (2022). Inflammation promotes resistance to immune checkpoint inhibitors in high microsatellite instability colorectal cancer. Nat Commun.

[50] Zou Z, Lin Z, Wu C, Tan J, Zhang J, Peng Y (2023). Micro-Engineered Organoid-on-a-Chip Based on Mesenchymal Stromal Cells to Predict Immunotherapy Responses of HCC Patients. Adv Sci (Weinh).

[51] Zanoni M, Cortesi M, Zamagni A, Arienti C, Pignatta S, Tesei A (2020). Modeling neoplastic disease with spheroids and organoids. J Hematol Oncol.

[52] Lee G, Suh Y, Park JY (2018). A Paired Bead and Magnet Array for Molding Microwells with Variable Concave Geometries. J Vis Exp.

[53] Song T, Kong B, Liu R, Luo Y, Wang Y, Zhao Y (2023). Bioengineering Approaches for the Pancreatic Tumor Organoids Research and Application. Adv Healthc Mater.

[54] Luo Z, Zhou X, Mandal K, He N, Wennerberg W, Qu M (2021). Reconstructing the tumor architecture into organoids. Adv Drug Deliver Rev.

[55] Kim S, Min S, Choi YS, Jo S, Jung JH, Han K (2022). Tissue extracellular matrix hydrogels as alternatives to Matrigel for culturing gastrointestinal organoids. Nat Commun.

[56] Costales-Carrera A, Fernandez-Barral A, Bustamante-Madrid P, Guerra L, Cantero R, Barbachano A (2019). Plocabulin Displays Strong Cytotoxic Activity in a Personalized Colon Cancer Patient-Derived 3D Organoid Assay. Mar Drugs.

[57] Yao Y, Xu X, Yang L, Zhu J, Wan J, Shen L (2020). Patient-Derived Organoids Predict Chemoradiation Responses of Locally Advanced Rectal Cancer. Cell Stem Cell.

[58] Shi X, Li Y, Yuan Q, Tang S, Guo S, Zhang Y (2022). Integrated profiling of human pancreatic cancer organoids reveals chromatin accessibility features associated with drug sensitivity. Nat Commun.

[59] Bi H, Ye K, Jin S (2020). Proteomic analysis of decellularized pancreatic matrix identifies collagen V as a critical regulator for islet organogenesis from human pluripotent stem cells. Biomaterials.

[60] van Tienderen GS, Rosmark O, Lieshout R, Willemse J, de Weijer F, Elowsson Rendin L (2023). Extracellular matrix drives tumor organoids toward desmoplastic matrix deposition and mesenchymal transition. Acta Biomater.

[61] Varinelli L, Guaglio M, Brich S, Zanutto S, Belfiore A, Zanardi F (2023). Decellularized extracellular matrix as scaffold for cancer organoid cultures of colorectal peritoneal metastases. J Mol Cell Biol.

[62] Ou L, Liu S, Wang H, Guo Y, Guan L, Shen L (2023). Patient-derived melanoma organoid models facilitate the assessment of immunotherapies. EBioMedicine.

[63] Mason BN, Starchenko A, Williams RM, Bonassar LJ, Reinhart-King CA (2013). Tuning three-dimensional collagen matrix stiffness independently of collagen concentration modulates endothelial cell behavior. Acta Biomater.

[64] Bordeleau F, Mason BN, Lollis EM, Mazzola M, Zanotelli MR, Somasegar S (2017). Matrix stiffening promotes a tumor vasculature phenotype. Proc Natl Acad Sci U S A.

[65] Ng S, Tan WJ, Pek MMX, Tan M, Kurisawa M (2019). Mechanically and chemically defined hydrogel matrices for patient-derived colorectal tumor organoid culture. Biomaterials.

[66] Tian YF, Ahn H, Schneider RS, Yang SN, Roman-Gonzalez L, Melnick AM (2015). Integrin-specific hydrogels as adaptable tumor organoids for malignant B and T cells. Biomaterials.

[67] Wu Y, Zhao Z, Guan Y, Zhang Y (2014). Galactosylated reversible hydrogels as scaffold for HepG2 spheroid generation. Acta Biomater.

[68] Atefi E, Lemmo S, Fyffe D, Luker GD, Tavana H (2014). High Throughput, Polymeric Aqueous Two-Phase Printing of Tumor Spheroids. Adv Funct Mater.

[69] Millet LJ, Gillette MU (2012). Over a century of neuron culture: from the hanging drop to microfluidic devices. Yale J Biol Med.

[70] Jørgensen A, Young J, Nielsen JE, Joensen UN, Toft BG, Rajpert-De Meyts E (2014). Hanging drop cultures of human testis and testis cancer samples: a model used to investigate activin treatment effects in a preserved niche. Br J Cancer.

[71] Kunz-Schughart LA, Kreutz M, Knuechel R (1998). Multicellular spheroids: a three-dimensional in vitro culture system to study tumour biology. Int J Exp Pathol.

[72] Lin RZ, Chang HY (2008). Recent advances in three-dimensional multicellular spheroid culture for biomedical research. Biotechnol J.

[73] Muhitch JW, O'Connor KC, Blake DA, Lacks DJ, Rosenzweig N, Spaulding GF (2000). Characterization of aggregation and protein expression of bovine corneal endothelial cells as microcarrier cultures in a rotating-wall vessel. Cytotechnology.

[74] Schneeberger K, Sánchez Romero N, Ye S, van Steenbeek FG, Oosterhoff LA, Pla Palacin I (2020). Large-Scale Production of LGR5-Positive Bipotential Human Liver Stem Cells. Hepatology.

[75] Yang Y, Huang R, Cao Z, Ma S, Chen D, Wang Z (2023). In vitro reconstitution of the hormone-responsive testicular organoids from murine primary testicular cells. Biofabrication.

[76] Yuhas JM, Li AP, Martinez AO, Ladman AJ (1977). A simplified method for production and growth of multicellular tumor spheroids. Cancer Res.

[77] Carvalho MP, Costa EC, Miguel SP, Correia IJ (2016). Tumor spheroid assembly on hyaluronic acid-based structures: A review. Carbohydr Polym.

[78] Azizipour N, Avazpour R, Sawan M, Ajji A, H RD (2022). Surface Optimization and Design Adaptation toward Spheroid Formation On-Chip. Sensors (Basel).

[79] Sun W, Zhang J, Qin Y, Tang H, Chen Y, Lin W (2022). A Simple and Efficient Strategy for Preparing a Cell-Spheroid-Based Bioink. Adv Healthc Mater.

[80] Guo W, Chen Z, Feng Z, Li H, Zhang M, Zhang H (2022). Fabrication of Concave Microwells and Their Applications in Micro-Tissue Engineering: A Review. Micromachines (Basel).

[81] Thomsen AR, Aldrian C, Bronsert P, Thomann Y, Nanko N, Melin N (2017). A deep conical agarose microwell array for adhesion independent three-dimensional cell culture and dynamic volume measurement. Lab Chip.

[82] Liu T, Chien CC, Parkinson L, Thierry B (2014). Advanced micromachining of concave microwells for long term on-chip culture of multicellular tumor spheroids. ACS Appl Mater Interfaces.

[83] Hu Y, Sui X, Song F, Li Y, Li K, Chen Z (2021). Lung cancer organoids analyzed on microwell arrays predict drug responses of patients within a week. Nat Commun.

[84] Jung YH, Choi D, Park K, Lee S, Kim J, Kim H (2021). Drug screening by uniform patient derived colorectal cancer hydro-organoids. Biomaterials.

[85] Clevers H (2016). Modeling Development and Disease with Organoids. Cell.

[86] Fan H, Demirci U, Chen P (2019). Emerging organoid models: leaping forward in cancer research. J Hematol Oncol.

[87] Park SE, Georgescu A, Huh D (2019). Organoids-on-a-chip. Science.

[88] Shirure VS, Bi Y, Curtis MB, Lezia A, Goedegebuure MM, Goedegebuure SP (2018). Tumor-on-a-chip platform to investigate progression and drug sensitivity in cell lines and patient-derived organoids. Lab Chip.

[89] Nashimoto Y, Mukomoto R, Imaizumi T, Terai T, Shishido S, Ino K (2023). Electrochemical sensing of oxygen metabolism for a three-dimensional cultured model with biomimetic vascular flow. Biosens Bioelectron.

[90] Xu Z, Li E, Guo Z, Yu R, Hao H, Xu Y (2016). Design and Construction of a Multi-Organ Microfluidic Chip Mimicking the in vivo Microenvironment of Lung Cancer Metastasis. ACS Appl Mater Interfaces.

[91] Fang G, Lu H, Al-Nakashli R, Chapman R, Zhang Y, Ju LA (2021). Enabling peristalsis of human colon tumor organoids on microfluidic chips. Biofabrication.

[92] Decante G, Costa JB, Silva-Correia J, Collins MN, Reis RL, Oliveira JM (2021). Engineering bioinks for 3D bioprinting. Biofabrication.

[93] Schwab A, Levato R, D Este M, Piluso S, Eglin D, Malda J (2020). Printability and Shape Fidelity of Bioinks in 3D Bioprinting. Chem Rev.

[94] Groll J, Burdick JA, Cho DW, Derby B, Gelinsky M, Heilshorn SC (2018). A definition of bioinks and their distinction from biomaterial inks. Biofabrication.

[95] Clark CC, Yoo KM, Sivakumar H, Strumpf K, Laxton AW, Tatter SB (2022). Immersion bioprinting of hyaluronan and collagen bioink-supported 3D patient-derived brain tumor organoids. Biomed Mater.

[96] Lee A, Hudson AR, Shiwarski DJ, Tashman JW, Hinton TJ, Yerneni S (2019). 3D bioprinting of collagen to rebuild components of the human heart. Science.

[97] Shi W, Mirza S, Kuss M, Liu B, Hartin A, Wan S (2023). Embedded Bioprinting of Breast Tumor Cells and Organoids Using Low-Concentration Collagen-Based Bioinks. Adv Healthc Mater.

[98] Enrico A, Voulgaris D, Östmans R, Sundaravadivel N, Moutaux L, Cordier A (2022). 3D Microvascularized Tissue Models by Laser-Based Cavitation Molding of Collagen. Adv Mater.

[99] Wu W, DeConinck A, Lewis JA (2011). Omnidirectional Printing of 3D Microvascular Networks. Adv Mater.

[100] Kolesky DB, Truby RL, Gladman AS, Busbee TA, Homan KA, Lewis JA (2014). 3D Bioprinting of Vascularized, Heterogeneous Cell-Laden Tissue Constructs. Adv Mater.

[101] Jia W, Gungor-Ozkerim PS, Zhang YS, Yue K, Zhu K, Liu W (2016). Direct 3D bioprinting of perfusable vascular constructs using a blend bioink. Biomaterials.

[102] Mollica PA, Booth-Creech EN, Reid JA, Zamponi M, Sullivan SM, Palmer X (2019). 3D bioprinted mammary organoids and tumoroids in human mammary derived ECM hydrogels. Acta Biomater.

[103] McGuckin C, Forraz N, Milet C, Lacroix M, Sbirkov Y, Sarafian V (2023). World's First Long-Term Colorectal Cancer Model by 3D Bioprinting as a Mechanism for Screening Oncolytic Viruses. Cancers (Basel).

[104] Yi H, Jeong YH, Kim Y, Choi Y, Moon HE, Park SH (2019). A bioprinted human-glioblastoma-on-a-chip for the identification of patient-specific responses to chemoradiotherapy. Nat Biomed Eng.

[105] Gunti S, Hoke ATK, Vu KP, London NR (2021). Organoid and Spheroid Tumor Models: Techniques and Applications. Cancers (Basel).

[106] Lee S, Koo I, Hwang HJ, Lee DW (2023). In Vitro three-dimensional (3D) cell culture tools for spheroid and organoid models. SLAS Discov.

[107] Bian S, Repic M, Guo Z, Kavirayani A, Burkard T, Bagley JA (2018). Genetically engineered cerebral organoids model brain tumor formation. Nat Methods.

[108] Hendriks D, Clevers H, Artegiani B (2020). CRISPR-Cas Tools and Their Application in Genetic Engineering of Human Stem Cells and Organoids. Cell Stem Cell.

[109] Momota H, Shih AH, Edgar MA, Holland EC (2008). c-Myc and beta-catenin cooperate with loss of p53 to generate multiple members of the primitive neuroectodermal tumor family in mice. Oncogene.

[110] Zhang S, Iyer S, Ran H, Dolgalev I, Gu S, Wei W (2021). Genetically Defined, Syngeneic Organoid Platform for Developing Combination Therapies for Ovarian Cancer. Cancer Discov.

[111] Lo Y, Kolahi KS, Du Y, Chang C, Krokhotin A, Nair A (2021). A CRISPR/Cas9-EngineeredARID1A-Deficient Human Gastric Cancer Organoid Model Reveals Essential and Nonessential Modes of Oncogenic Transformation. Cancer Discov.

[112] Thege FI, Rupani DN, Barathi BB, Manning SL, Maitra A, Rhim AD (2022). A Programmable In Vivo CRISPR Activation Model Elucidates the Oncogenic and Immunosuppressive Functions of MYC in Lung Adenocarcinoma. Cancer Res.

[113] Takebe T, Wells JM (2019). Organoids by design. Science.

[114] Murrow LM, Weber RJ, Gartner ZJ (2017). Dissecting the stem cell niche with organoid models: an engineering-based approach. Development.

[115] Centenera MM, Raj GV, Knudsen KE, Tilley WD, Butler LM (2013). Ex vivo culture of human prostate tissue and drug development. Nat Rev Urol.

[116] Tentler JJ, Tan AC, Weekes CD, Jimeno A, Leong S, Pitts TM (2012). Patient-derived tumour xenografts as models for oncology drug development. Nat Rev Clin Oncol.

[117] Fujii M, Shimokawa M, Date S, Takano A, Matano M, Nanki K (2016). A Colorectal Tumor Organoid Library Demonstrates Progressive Loss of Niche Factor Requirements during Tumorigenesis. Cell Stem Cell.

[118] Mo S, Tang P, Luo W, Zhang L, Li Y, Hu X (2022). Patient-Derived Organoids from Colorectal Cancer with Paired Liver Metastasis Reveal Tumor Heterogeneity and Predict Response to Chemotherapy. Adv Sci.

[119] Yan H, Siu HC, Law S, Ho SL, Yue S, Tsui WY (2018). A Comprehensive Human Gastric Cancer Organoid Biobank Captures Tumor Subtype Heterogeneity and Enables Therapeutic Screening. Cell Stem Cell.

[120] Lee SH, Hu W, Matulay JT, Silva MV, Owczarek TB, Kim K (2018). Tumor Evolution and Drug Response in Patient-Derived Organoid Models of Bladder Cancer. Cell.

[121] Calandrini C, Schutgens F, Oka R, Margaritis T, Candelli T, Mathijsen L (2020). An organoid biobank for childhood kidney cancers that captures disease and tissue heterogeneity. Nat Commun.

[122] Jacob F, Salinas RD, Zhang DY, Nguyen P, Schnoll JG, Wong S (2020). A Patient-Derived Glioblastoma Organoid Model and Biobank Recapitulates Inter- and Intra-tumoral Heterogeneity. Cell.

[123] Kawasaki K, Toshimitsu K, Matano M, Fujita M, Fujii M, Togasaki K (2020). An Organoid Biobank of Neuroendocrine Neoplasms Enables Genotype-Phenotype Mapping. Cell.

[124] Zhang J, Chen B, Li H, Wang Y, Liu X, Wong KY (2023). Cancer-associated fibroblasts potentiate colorectal cancer progression by crosstalk of the IGF2-IGF1R and Hippo-YAP1 signaling pathways. J Pathol.

[125] Lam YK, Yu J, Huang H, Ding X, Wong AM, Leung HH (2023). TP53 R249S mutation in hepatic organoids captures the predisposing cancer risk. Hepatology.

[126] Barretina J, Caponigro G, Stransky N, Venkatesan K, Margolin AA, Kim S (2012). The Cancer Cell Line Encyclopedia enables predictive modelling of anticancer drug sensitivity. Nature.

[127] Wong CH, Siah KW, Lo AW (2019). Estimation of clinical trial success rates and related parameters. Biostatistics.

[128] Gao H, Korn JM, Ferretti S, Monahan JE, Wang Y, Singh M (2015). High-throughput screening using patient-derived tumor xenografts to predict clinical trial drug response. Nat Med.

[129] Kopper O, de Witte CJ, Lohmussaar K, Valle-Inclan JE, Hami N, Kester L (2019). An organoid platform for ovarian cancer captures intra- and interpatient heterogeneity. Nat Med.

[130] Vlachogiannis G, Hedayat S, Vatsiou A, Jamin Y, Fernandez-Mateos J, Khan K (2018). Patient-derived organoids model treatment response of metastatic gastrointestinal cancers. Science.

[131] Kondo J, Ekawa T, Endo H, Yamazaki K, Tanaka N, Kukita Y (2019). High-throughput screening in colorectal cancer tissue-originated spheroids. Cancer Sci.

[132] Srimongkol A, Laosillapacharoen N, Saengwimol D, Chaitankar V, Rojanaporn D, Thanomchard T (2023). Sunitinib efficacy with minimal toxicity in patient-derived retinoblastoma organoids. J Exp Clin Cancer Res.

[133] Wang H, Zhang C, Peng K, Chen Z, Su J, Li Y (2023). Using patient-derived organoids to predict locally advanced or metastatic lung cancer tumor response: A real-world study. Cell Rep Med.

[134] Kang YA, Deng J, Ling J, Li X, Chiang Y, Koay EJ (2022). 3D imaging analysis on an organoid-based platform guides personalized treatment in pancreatic ductal adenocarcinoma. J Clin Invest.

[135] Oh DY, Lee KH, Lee DW, Yoon J, Kim TY, Bang JH (2022). Gemcitabine and cisplatin plus durvalumab with or without tremelimumab in chemotherapy-naive patients with advanced biliary tract cancer: an open-label, single-centre, phase 2 study. Lancet Gastroenterol Hepatol.

[136] Ren X, Huang M, Weng W, Xie Y, Wu Y, Zhu S (2023). Personalized drug screening in patient-derived organoids of biliary tract cancer and its clinical application. Cell Rep Med.

[137] Tempero MA, Malafa MP, Al-Hawary M, Behrman SW, Benson AB, Cardin DB (2021). Pancreatic Adenocarcinoma, Version 2.2021, NCCN Clinical Practice Guidelines in Oncology. J Natl Compr Canc Netw.

[138] Lv T, Shen L, Xu X, Yao Y, Mu P, Zhang H (2023). Patient-derived tumor organoids predict responses to irinotecan-based neoadjuvant chemoradiotherapy in patients with locally advanced rectal cancer. Int J Cancer.

[139] Fathi A, Christians KK, George B, Ritch PS, Erickson BA, Tolat P (2015). Neoadjuvant therapy for localized pancreatic cancer: guiding principles. J Gastrointest Oncol.

[140] Demyan L, Habowski AN, Plenker D, King DA, Standring OJ, Tsang C (2022). Pancreatic Cancer Patient-derived Organoids Can Predict Response to Neoadjuvant Chemotherapy. Ann Surg.

[141] Hsu K, Adileh M, Martin ML, Makarov V, Chen J, Wu C (2022). Colorectal Cancer Develops Inherent Radiosensitivity That Can Be Predicted Using Patient-Derived Organoids. Cancer Res.

[142] Ganesh K, Wu C, O Rourke KP, Szeglin BC, Zheng Y, Sauvé CG (2019). A rectal cancer organoid platform to study individual responses to chemoradiation. Nat Med.

[143] Zeng Y, Li S, Zhang S, Wang L, Yuan H, Hu F (2022). Cell membrane coated-nanoparticles for cancer immunotherapy. Acta Pharm Sin B.

[144] Waldman AD, Fritz JM, Lenardo MJ (2020). A guide to cancer immunotherapy: from T cell basic science to clinical practice. Nat Rev Immunol.

[145] Sharma P, Allison JP (2015). The future of immune checkpoint therapy. Science.

[146] Scognamiglio G, De Chiara A, Parafioriti A, Armiraglio E, Fazioli F, Gallo M (2019). Patient-derived organoids as a potential model to predict response to PD-1/PD-L1 checkpoint inhibitors. Brit J Cancer.

[147] Qin L, Wang L, Zhang J, Zhou H, Yang Z, Wang Y (2022). Therapeutic strategies targeting uPAR potentiate anti-PD-1 efficacy in diffuse-type gastric cancer. Sci Adv.

[148] Wang W, Yuan T, Ma L, Zhu Y, Bao J, Zhao X (2022). Hepatobiliary Tumor Organoids Reveal HLA Class I Neoantigen Landscape and Antitumoral Activity of Neoantigen Peptide Enhanced with Immune Checkpoint Inhibitors. Adv Sci (Weinh).

[149] LeSavage BL, Suhar RA, Broguiere N, Lutolf MP, Heilshorn SC (2022). Next-generation cancer organoids. Nat Mater.

[150] Veninga V, Voest EE (2021). Tumor organoids: Opportunities and challenges to guide precision medicine. Cancer Cell.

[151] Ooft SN, Weeber F, Schipper L, Dijkstra KK, McLean CM, Kaing S (2021). Prospective experimental treatment of colorectal cancer patients based on organoid drug responses. ESMO Open.

[152] He A, Huang Y, Cheng W, Zhang D, He W, Bai Y (2020). Organoid culture system for patient-derived lung metastatic osteosarcoma. Med Oncol.

[153] Aisenbrey EA, Murphy WL (2020). Synthetic alternatives to Matrigel. Nat Rev Mater.

[154] Hughes CS, Postovit LM, Lajoie GA (2010). Matrigel: a complex protein mixture required for optimal growth of cell culture. Proteomics.

[155] Willert K, Brown JD, Danenberg E, Duncan AW, Weissman IL, Reya T (2003). Wnt proteins are lipid-modified and can act as stem cell growth factors. Nature.

[156] Loebel C, Weiner AI, Eiken MK, Katzen JB, Morley MP, Bala V (2022). Microstructured Hydrogels to Guide Self-Assembly and Function of Lung Alveolospheres. Adv Mater.

[157] Liu H, Wang Y, Wang H, Zhao M, Tao T, Zhang X (2020). A Droplet Microfluidic System to Fabricate Hybrid Capsules Enabling Stem Cell Organoid Engineering. Adv Sci.

[158] Zhang Q, Wang X, Kuang G, Yu Y, Zhao Y (2022). Photopolymerized 3D Printing Scaffolds with Pt (IV) Prodrug Initiator for Postsurgical Tumor Treatment. Research (Wash D C).

[159] Zhang Q, Wang X, Kuang G, Zhao Y (2022). Pt (IV) prodrug initiated microparticles from microfluidics for tumor chemo-, photothermal and photodynamic combination therapy. Bioact Mater.

[160] Jin MZ, Han RR, Qiu GZ, Ju XC, Lou G, Jin WL (2018). Organoids: An intermediate modeling platform in precision oncology. Cancer Lett.

[161] Wang J, Huang D, Yu H, Cheng Y, Ren H, Zhao Y (2022). Developing tissue engineering strategies for liver regeneration. Engineered Regeneration.

[162] Clinton J, McWilliams-Koeppen P (2019). Initiation, Expansion, and Cryopreservation of Human Primary Tissue-Derived Normal and Diseased Organoids in Embedded Three-Dimensional Culture. Curr Protoc Cell Biol.

[163] Zhang Q, Kuang G, Yu Y, Ding X, Ren H, Sun W (2022). Hierarchical Microparticles Delivering Oxaliplatin and NLG919 Nanoprodrugs for Local Chemo-immunotherapy. ACS Appl Mater Interfaces.

[164] Hospodiuk M, Dey M, Sosnoski D, Ozbolat IT (2017). The bioink: A comprehensive review on bioprintable materials. Biotechnol Adv.

[165] Jang J, Kim TG, Kim BS, Kim SW, Kwon SM, Cho DW (2016). Tailoring mechanical properties of decellularized extracellular matrix bioink by vitamin B2-induced photo-crosslinking. Acta Biomater.

[166] Pourchet LJ, Thepot A, Albouy M, Courtial EJ, Boher A, Blum LJ (2017). Human Skin 3D Bioprinting Using Scaffold-Free Approach. Adv Healthc Mater.

[167] Wang Y, Ma X, Zhou W, Liu C, Zhang H (2022). Reregulated mitochondrial dysfunction reverses cisplatin resistance microenvironment in colorectal cancer. Smart Medicine.

[168] Meng F, Meyer CM, Joung D, Vallera DA, McAlpine MC, Panoskaltsis-Mortari A (2019). 3D Bioprinted In Vitro Metastatic Models via Reconstruction of Tumor Microenvironments. Adv Mater.

[169] Huang D, Zhang X, Fu X, Zu Y, Sun W, Zhao Y (2021). Liver spheroids on chips as emerging platforms for drug screening. Engineered Regeneration.

[170] Nothdurfter D, Ploner C, Coraça-Huber DC, Wilflingseder D, Müller T, Hermann M (2022). 3D bioprinted, vascularized neuroblastoma tumor environment in fluidic chip devices for precision medicine drug testing. Biofabrication.

[171] Wadman M (2023). FDA no longer has to require animal testing for new drugs. Science.

